# Gut microbiota, nutrients, and depression

**DOI:** 10.3389/fnut.2025.1581848

**Published:** 2025-10-15

**Authors:** Yajun Qiao, Lin Rong, Hanxi Chen, Juan Guo, Guoqiang Li, Qiannan Wang, Hongtao Bi, Lixin Wei, Tingting Gao

**Affiliations:** ^1^School of Psychology, Chengdu Medical College, Chengdu, China; ^2^Qinghai Provincial Key Laboratory of Tibetan Medicine Pharmacology and Safety Evaluation, Northwest Institute of Plateau Biology, Chinese Academy of Sciences, Xining, China; ^3^Department of Psychiatry, The People's Hospital of Jiangmen, Southern Medical University, Jiangmen, China; ^4^University of Chinese Academy of Sciences, Beijing, China; ^5^CAS Key Laboratory of Tibetan Medicine Research, Northwest Institute of Plateau Biology, Chinese Academy of Sciences, Xining, China

**Keywords:** diet, depression, gut microbiota, nutrients, brain-gut axis

## Abstract

In the post-COVID-19 era, depression incidence has risen sharply, and a healthy diet is confirmed to lower this risk. However, two critical gaps remain: it is unclear whether nutrients alleviate depressive symptoms by improving the gut microbiota, and existing evidence has notable limitations. This study aimed to address these by exploring how deficiencies in key nutrients (protein, lipids, sugars, vitamins, and minerals) affect gut microbiota diversity—potentially a driver of early depression—and systematically evaluating clinical/basic research on nutrients' role in gut microbiota-mediated depression intervention. Results showed nutrients enhance gut microbiota abundance and diversity, regulate the gut-brain axis to boost short-chain fatty acid (SCFA) and neurotransmitter synthesis, and reduce inflammation, thereby alleviating depression. Thus, a healthy anti-inflammatory diet (rich in vegetables, fruits, fish) may lower depressive symptom risk. Three key research gaps were identified: 1. Mechanistic evidence relies heavily on animal studies (e.g., mouse neurotransmitter experiments) with insufficient large-scale human randomized controlled trials (RCTs) to confirm causality; 2. Conflicting findings exist [e.g., alpha-linolenic acid (ALA) has no antidepressant effect in some human cohorts]; 3. The dose-response relationship (e.g., fiber needed to elevate SCFAs to antidepressant levels) is unquantified. Future studies should quantify dietary patterns and target gut microbiota metabolism to advance early depression prevention and deepen understanding of diet-microbiota-depression links.

## Graphical Abstract

This figure elucidates the intricate relationship between diet, gut microbiota, and depressed mood. It demonstrates that various dietary nutrients, including plant- and animal-based proteins, fats, and diverse mineral elements, influence depressive mood primarily through post-ingestive regulation of gut microbiota. This occurs via three key mechanisms: modulation of short-chain fatty acid (SCFA) production, inflammation, and neurotransmitter levels. The illustration underscores the complex physiological interplay between diet, gut microbiota, and mood, offering novel insights into the underlying mechanisms of depression. These findings suggest that dietary modification and manipulation of gut microbiota may represent promising intervention strategies for alleviating depressive symptoms. 
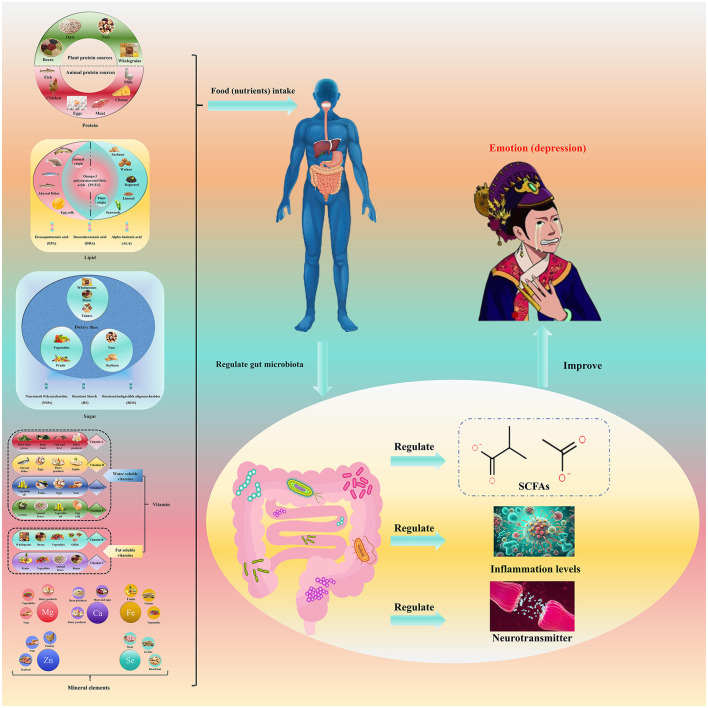

## Highlights

- This paper systematically reviews the relationship between nutrition, microbiota, and depression.
- This paper proposes that microbial composition can be modulated by dietary nutrients and improve depression-like symptoms.
- The review used the gut microbiota to establish links between dietary factors and mental disorders.
- From the perspective of nutrition, this paper provides the basis for diet prevention and early intervention of depression.

## 1 Introduction

At present, depression has become a major mental illness worldwide (1), especially since the COVID-19 pandemic, when the incidence of depression has accelerated by 25% globally (2, 3). This review focuses on “major depressive disorder (MDD)”—the most common subtype characterized by persistent low mood, loss of interest, and impairment in social/occupational function—rather than bipolar depression or situational depression, as MDD has the strongest evidence linking to dietary and gut microbiota changes (1, 4). According to the Global Burden of Disease Study 2023 [consistent with (2) but more precise], MDD affects approximately 280 million people globally, accounting for 3.6% of the total population; in low- and middle-income countries, the 12-month prevalence of MDD reaches 5.9% (range: 3.8%−10.4%) (5), and post-COVID-19, the incidence in adolescents and young adults (18–25 years) increased by 40% compared with pre-pandemic levels (2). However, existing medical methods (e.g., antidepressants) have a response rate of only 50% in MDD patients (6), and early clinical symptoms of depression (e.g., anhedonia, sleep disturbance) are often ignored, further worsening health outcomes. However, existing medical methods cannot be used to diagnose and prevent depression early, and early clinical symptoms of depression affect the health and function of the human body. Therefore, new approaches are needed for early intervention and prevention of depression. Recent studies have shown that nutritional deficiencies are closely related to mental health (7–9), and adhering to healthy dietary patterns (e.g., Mediterranean diet) can reduce depression risk—yet these patterns exert effects primarily through their core nutrients (e.g., omega-3 from fish, fiber from vegetables) (10). We focus on nutrient deficiency rather than “diet as a whole” for three reasons: 1. functional specificity: diet is a complex mixture of components, while nutrients (e.g., protein, vitamin D) are the functional units that directly interact with gut microbiota and regulate physiological processes linked to depression (e.g., neurotransmitter synthesis, inflammation) (11, 12); 2. causal relevance: nutrient deficiency (e.g., tryptophan shortage) is a modifiable risk factor for early depression, whereas “unhealthy diet” is a broad concept that includes non-nutritional factors (e.g., food processing) (7, 9); 3. mechanistic clarity: the link between nutrient deficiency, gut microbiota dysbiosis (e.g., reduced *Bifidobacterium*), and depressive symptoms is more directly measurable (e.g., via SCFA levels, neurotransmitter concentrations) than the vague association between “overall diet” and mood (11, 13). However, owing to the complex relationship between mood and eating habits, people choose some favorite junk food when they are depressed, such as high-fat and high-sugar food (french fries, soda, and fried food); while so-called “comfort food” (e.g., hot pot) and stimulating food (e.g., spicy snacks, processed meats)—the latter share similar high-fat/high-salt traits to the aforementioned unhealthy foods; although these foods can temporarily regulate mood, long-term consumption affects health. Therefore, it is difficult to explain the mechanism by which nutrition regulates mood. This complexity stems from three key aspects: 1. bidirectional interaction between mood and diet: depressive mood may reduce intake of nutrient-dense foods (e.g., vegetables, fish) and increase craving for junk food, forming a “malnutrition-depression” cycle that confounds causal inference (14, 15); 2. interindividual variability: gut microbiota composition (e.g., Bifidobacterium abundance) and nutrient metabolism capacity (e.g., omega-3 conversion efficiency) differ by age, ethnicity, and lifestyle, leading to heterogeneous responses to nutritional intervention (16, 17); 3. multilevel mediation of the gut-brain axis: Nutrients act on mood not directly, but via gut microbiota-derived metabolites (e.g., SCFAs), neurotransmitter synthesis (e.g., tryptophan → 5-HT), and immune inflammation regulation—these cascading pathways are difficult to disentangle in human studies (13, 18).

The gut-brain axis—a bidirectional communication network involving the central nervous system (CNS), enteric nervous system (ENS), and gut microbiota—provides a key framework for understanding “nutrition-microbiota-depression” interactions (13, 18). Specifically, the gut microbiota acts as a “metabolic bridge”: it ferment dietary nutrients to produce bioactive metabolites (e.g., SCFAs, tryptophan derivatives), which signal to the brain via three pathways: 1. circulation [e.g., SCFAs enter the bloodstream and cross the blood-brain barrier (19)]; 2. vagus nerve [ENS sensory neurons transmit microbiota-derived signals to the CNS (20)]; 3. immune system [microbiota regulate systemic inflammation, which affects brain function (21, 22)]. Disruption of this axis (e.g., gut dysbiosis reducing SCFA production) is closely associated with MDD, as shown by reduced SCFA levels and altered microbiota composition (e.g., decreased *Bifidobacterium*) in depressed patients (23, 24). In addition, the gut is the core part of the body's nutrient absorption system, and the microbiota is also involved in the metabolic process of nutrition (13). Interestingly, the microbiota in the gut can also intervene in the development of depression (25). The metabolites of the gut microbiota may affect the synthesis of neurotransmitters through the tryptophan metabolism pathway and interfere with the brain's regulation of emotions (18). However, there is currently a lack of research on the correlations among depression, the gut microbiota and nutrition. Therefore, in this study, we focused on the impact of nutrient deficiency (proteins, lipids, sugars, vitamins and minerals) on the diversity of the gut microbiota, which may be one of the conditions underlying the early occurrence of depression (7–9, 11).

## 2 Methods

### 2.1 Literature search strategy

This review followed a systematic approach aligned with the Preferred Reporting Items for Systematic Reviews and Meta-Analyses (PRISMA) guidelines (26).

- **Search databases:** a systematic search will be conducted across four major databases: PubMed, Web of Science Core Collection, Embase, and Cochrane Library. Additionally, the China National Knowledge Infrastructure (CNKI) database will be included to ensure comprehensive coverage of relevant Chinese literature.
- **Keywords and search terms:** the search strategy will integrate both subject headings and free-text terms. Core keywords include “dietary fiber,” “gut inflammation,” “depression,” “gut microbiota,” “short-chain fatty acids,” “omega-3 polyunsaturated fatty acids,” “vitamins,” and “minerals.” Boolean operators (AND/OR) will be used to construct comprehensive search expressions, such as “(dietary fiber OR resistant starch) AND (gut inflammation OR intestinal barrier) AND (depression OR mood disorder).”
- **Time period:** studies published between January 2013 and December 2023 will be included to ensure the retrieval of the most recent evidence within the past decade. Foundational studies published prior to 2013, such as those focusing on SCFAs and the brain-gut axis, will also be considered.
- **Language restriction:** only peer-reviewed full-text articles written in either Chinese or English will be included. Abstracts, conference proceedings, and non-peer-reviewed materials will be excluded.

### 2.2 Study selection criteria

- **Inclusion criteria:**
  1. study types: randomized controlled trials (RCTs), cohort studies, case-control studies, and animal experiments with clearly defined model construction methods;
  2. Outcome indicators: studies reporting any association related to the pathway “nutrients [e.g., dietary fiber, omega-3 polyunsaturated fatty acids (PUFAs)] → intestinal inflammation → depression,” including but not limited to intestinal inflammation markers [e.g., interleukin-6 (IL-6), Tumor necrosis factor-α (TNF-α)], depression assessment scores [e.g., Beck Depression Inventory-II (BDI-II), Patient Health Questionnaire-9 (PHQ-9)], and changes in gut microbiota composition;
  3. Sample size: human studies must include at least 50 participants, while animal studies must have a minimum of six animals per group.
- **Exclusion criteria:**
  1. Review articles, meta-analyses, and commentaries;
  2. Studies involving patients with severe organic diseases (e.g., cancer, chronic kidney disease) to avoid confounding effects of underlying conditions on inflammation or depression outcomes;
  3. Studies with incomplete data or those lacking extractable key outcome measures.

## 3 Proteins, gut microbiota, and depression

### 3.1 Association between protein intake and depression

Adequate protein intake is essential for human growth, development, and health maintenance (12), and it is also associated with the prevalence of depression (27–30). However, current research on the relationship between protein sources and depression risk remains limited. Existing studies have only confirmed two key findings: first, milk and plant-derived proteins can reduce the incidence of depression (29); second, red meat and processed meat may increase the incidence of depression (30). The mood-beneficial effect of milk/dairy and plant proteins stems from their “high biological value”: they contain complete essential amino acids (e.g., tryptophan, tyrosine) that are critical for neurotransmitter synthesis—tryptophan is the precursor of 5-hydroxytryptamine (5-HT, a key mood-regulating neurotransmitter), and tyrosine is the precursor of dopamine (related to motivation and pleasure) (31). In contrast, red meat/processed meat has lower tryptophan content and may induce gut microbiota dysbiosis [e.g., elevated Bacteroides (23)], which exacerbates inflammation and depressive symptoms (30). In addition, the impact of individual differences in eating habits on mood has rarely been studied. These research limitations further complicate the effort to clarify the relationship between protein intake and depression. Therefore, this section focuses on exploring the beneficial effects of protein intake.

It is necessary to emphasize the synergistic role of both “quality” and “quantity” when examining protein's regulation of depression: 1. in terms of quality, the antidepressant effect of high-biological-value proteins (containing complete essential amino acids, such as dairy and fish proteins) is significantly superior to that of low-biological-value proteins (such as single-grain proteins). A cohort study of American adults (27) showed that individuals who consumed high-biological-value proteins (accounting for more than 50% of total protein intake) daily had a 28% lower risk of depression (OR = 0.72, 95% CI: 0.58–0.89), while low-biological-value proteins did not provide such a protective effect. This is directly related to the higher content of tryptophan and tyrosine in high-biological-value proteins, which support neurotransmitter synthesis (31); 2. in terms of quantity, there is a “threshold effect”: a study of Indian middle school students (28) indicated that when daily protein intake was ≥1.2 g/kg body weight, intestinal tryptophan supply was stable, 5-HT (5-hydroxytryptamine) synthesis was sufficient, and depression scores decreased significantly (mean difference = −1.8 points, *P* < 0.01); intake below this threshold increased the risk of depression, while excessive intake (≥2.0 g/kg body weight) did not further enhance the antidepressant effect. Instead, the increased metabolic burden of protein led to a higher abundance of intestinal *Bacteroides* (23), which may induce microbiota dysbiosis. Additionally, tryptophan supplementation (500 mg/day) can temporarily improve mood in patients with mild depression (reducing BDI-II scores by 2.5 points), but high doses (≥1,000 mg/day) may induce serotonin syndrome due to excessive activation of 5-HT (31); tyrosine supplementation (1,000 mg/day) is effective for depressed patients with fatigue symptoms (increasing energy scores by 15%), which is related to tyrosine acting as a dopamine precursor to improve motivation (32); in contrast, glutamine supplementation (2,000 mg/day) showed no antidepressant effect due to its low blood-brain barrier penetration rate (< 10%) (33). These results suggest that single amino acids need to be applied precisely to specific depression subtypes (such as fatigue-type or low-5-HT-type depression) rather than being used as a broad-spectrum intervention.

### 3.2 Material basis for proteins influencing depression: amino acids and neurotransmitters

Proteins are macromolecules composed of one or more long amino acid chains, and most neurotransmitters are amino acid derivatives. This material connection provides a key biological basis for proteins to influence depression:

- Tryptophan and tyrosine are precursor substances of serotonin, dopamine, and norepinephrine, respectively (34, 35);
- Glutamate is the precursor of gamma-aminobutyric acid (GABA), an inhibitory neurotransmitter (33).

In terms of food sources, tryptophan and tyrosine are widely distributed in different types of foods:

- Tryptophan is abundant in plant-based foods (e.g., legumes, oats, nuts, and whole grains) (32, 36);
- Tyrosine is rich in animal-based foods (e.g., milk, cheese, meat, eggs, chicken, and fish) (32, 36).

Based on the aforementioned material connections, studies have found that reduced protein intake in elderly mice leads to abnormal neurotransmitter levels and impairments in cognitive and behavioral functions (37). This result further confirms the close link between protein intake, neural function, and mood regulation.

### 3.3 Two mechanisms by which proteins influence mood

**(1) Tryptophan-serotonin pathway: regulating neurotransmitter synthesis**

Notably, tryptophan entry into the CNS is competitively regulated by L-amino acid transporter 1 (LAT1), which is shared with branched-chain amino acids (BCAAs, e.g., leucine, isoleucine). When dietary BCAA intake is high, they occupy LAT1, reducing tryptophan uptake by the brain and subsequently decreasing 5-HT synthesis (31, 38). Additionally, tryptophan hydroxylase exists in two isoforms: Tryptophan hydroxylase 1 (TPH1; predominant in gut enterochromaffin cells) and tryptophan hydroxylase 2 (TPH2; specific to CNS neurons). TPH2 activity in the brain is the rate-limiting step for 5-HT synthesis, and its expression is downregulated by chronic stress—an effect reversed by adequate tryptophan intake (31, 39). Serotonin is a potential biological marker for depression; a decrease in serotonin levels in the body can induce anxiety and depressive symptoms (40–42). Notably, 90% of the body's serotonin is produced by intestinal enterochromaffin cells (ECs) (39), but this intestinal serotonin cannot cross the blood-brain barrier (BBB). The BBB expresses L-amino acid transporter 1 (LAT1), which preferentially transports branched-chain amino acids (BCAAs) over serotonin—preventing intestinal serotonin from entering the CNS (31). Instead, CNS serotonin is synthesized *de novo* from tryptophan that crosses the BBB via LAT1 (when BCAA competition is low) (31, 39). This indicates that protein intake can regulate the synthesis of serotonin by controlling tryptophan supply, thereby participating in the process of mood regulation.

**(2) Gut microbiota-mediated protein metabolism: bidirectional regulation of depression-related substances**

The gut is the core site for protein absorption and transformation, and gut microbiota, as a key component of nutrient absorption, participates in the absorption, metabolism, and transformation of dietary proteins in the gastrointestinal tract. It mediates the effects of proteins on depression through two pathways:

- Regulating intestinal serotonin synthesis

ECs in the gut are major epithelial chemical sensors and can produce more than 90% of the serotonin in the human body (39). SCFAs exert their mood-regulating effects primarily through activating G protein-coupled receptors (GPRs) and inhibiting histone deacetylases (HDACs). G protein-coupled receptors 41 (GPR41) and G protein-coupled receptors 43 (GPR43; expressed on intestinal epithelial cells and immune cells) are activated by acetic acid and propionic acid, triggering downstream signaling that upregulates the expression of tight junction proteins [e.g., occludin, zonula occludens-1 (ZO-1)] to enhance intestinal barrier integrity (43, 44). Butyric acid, in particular, acts as a potent histone deacetylase (HDAC) inhibitor in colonocytes and CNS neurons: it increases histone acetylation at the promoter of the BDNF (brain-derived neurotrophic factor) gene, promoting BDNF transcription—BDNF is critical for neuronal survival and synaptic plasticity, and its downregulation is linked to depression (23, 43).

Notably, both these protein-fermenting microbiota and their metabolites (e.g., SCFAs) are closely associated with the development of depression (45, 46). For example, the abundance of *Bacteroides* increases in the gut of depressed patients, while the abundances of *Bifidobacterium, Lactobacillus*, and *Ruminococcus* decrease (23). The association between these microorganisms and depression is determined by their metabolic functions:

- *Bacteroides* (increased in depression): excessive *Bacteroides* accelerates abnormal protein fermentation, producing pro-inflammatory metabolites (e.g., indole, p-cresol) that disrupt the intestinal barrier and increase LPS-induced inflammation—this exacerbates depressive symptoms via the gut-brain axis (23, 30);
- *Bifidobacterium*/*Lactobacillus* (decreased in depression): these are core SCFA-producing bacteria (acetate/propionate) and can upregulate intestinal serotonin synthesis (23, 43). Their reduction leads to SCFA deficiency and impaired gut barrier, weakening neuroprotective and anti-inflammatory effects (20, 23);
- *Ruminococcus* (decreased in depression): as a key butyrate-producing bacterium, *Ruminococcus* supports hippocampal BDNF expression via butyrate (43). Its deficiency reduces butyrate levels, compromising neuroplasticity and mood regulation (23).

- Fermenting proteins to produce SCFAs

After dietary protein intake, specific gut microbiota (e.g., *Bifidobacterium, Lactobacillus, Bacteroides, Roseburia, Coprococcus*, and *Ruminococcus*) can ferment the protein to produce short-chain fatty acids, mainly including acetic acid, propionic acid, and butyric acid (43, 47).

Notably, both these protein-fermenting microbiota and their metabolites (SCFAs) are closely associated with the development of depression (45, 46). For example, the abundance of Bacteroides increases in the gut of depressed patients, while the abundances of *Bifidobacterium, Lactobacillus*, and *Ruminococcus* decrease (23).

### 3.4 The association between proteins, gut microbiota, and depression

**Figure 1:**
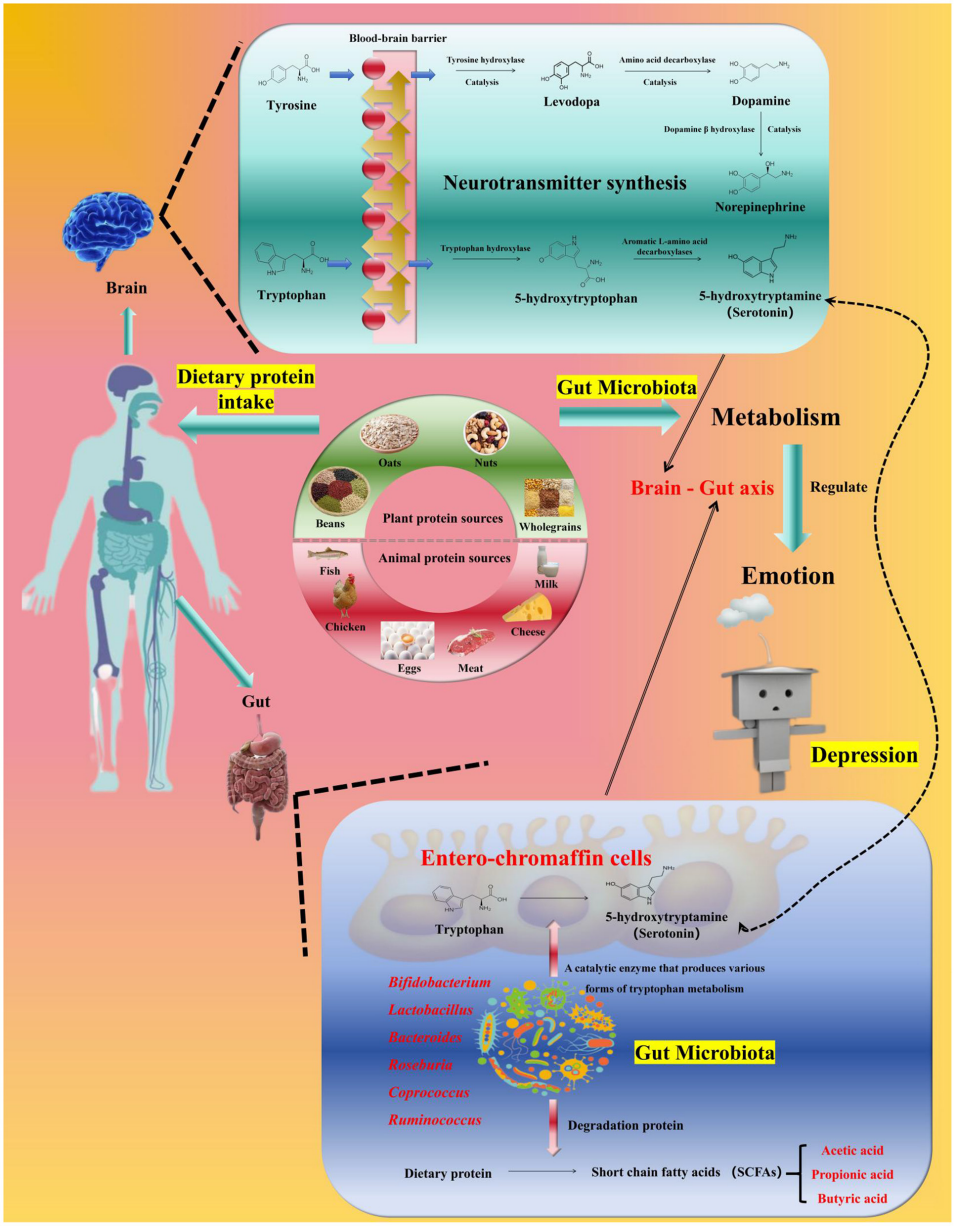
Relationships among protein, the gut microbiota, and mood. Dietary protein (milk/plant-derived protein preferred) is fermented by gut microbiota (e.g., *Bifidobacterium, Lactobacillus*) to produce short-chain fatty acids (SCFAs) and regulate tryptophan metabolism—tryptophan enters the brain to synthesize 5-hydroxytryptamine (5-HT), while SCFAs enhance intestinal barrier function and promote brain-derived neurotrophic factor (BDNF) expression via the gut-brain axis. In contrast, excessive red/processed meat increases *Bacteroides*, inducing inflammation and exacerbating depression. Key bacterial groups and their functions are labeled to clarify the regulatory cascade.

In summary, there is a close association between protein absorption, gut microbiota, and depression, which is primarily achieved through the following two mechanisms:

- Influencing the synthesis of neurotransmitters (e.g., serotonin) via the tryptophan metabolism pathway;
- Metabolites (e.g., SCFAs) produced by gut microbiota during protein metabolism and absorption participating in the regulation of depression;

The functions of gut microbiota directly involved in the development of depression (see Figure 1).

### 3.5 Association between protein intake and depression, and research limitations

However, the current evidence has critical limitations that require balanced interpretation, including conflicting findings, overreliance on observational data, and unclear causality.

- First, animal studies predominate over human clinical data: the mechanistic link between protein deficiency and neurotransmitter abnormalities relies heavily on elderly mouse experiments (37), but human depression involves psychosocial factors (e.g., stress, social isolation) absent in animal models, and mouse neurotransmitter systems (e.g., 5-HT turnover rate) differ from humans, limiting translational value (37).
- Second, observational studies have inherent methodological flaws: the cited surveys (27, 28) are cross-sectional or prospective observational designs, which cannot rule out reverse causation (e.g., depressed patients may reduce dairy intake due to appetite loss) or confounding factors [e.g., high dairy consumers often have healthier lifestyles, such as regular physical activity, not fully adjusted in (27, 28)].
- Finally, correlation does not equal causation: the original analysis implies protein intake “reduces depression risk,” but current data only confirm an association—no RCT has directly validated that increasing protein intake alleviates depressive symptoms in humans (27, 28).

## 4 Omega-3 polyunsaturated fatty acids, the gut microbiota, and depression

### 4.1 Classification and main sources of dietary fats

Lipids are essential macronutrients, and their dietary sources relevant to depression intervention primarily include:

- **Plant-derived unsaturated fatty acids:** soybeans, wheat germ, and certain vegetable oils are sources of alpha-linolenic acid [ALA, a precursor of omega-3 polyunsaturated fatty acids (PUFAs)] (48–52);
- **Animal-derived omega-3 PUFAs:** deep-sea fish (e.g., salmon, mackerel) are excellent sources of eicosapentaenoic acid (EPA) and docosahexaenoic acid (DHA)—the two most biologically active omega-3 subtypes for mood regulation (5);
- **Auxiliary lipid sources:** egg yolks and liver contain phospholipids, which support omega-3 absorption and transport (53).

### 4.2 The link between lipids, brain function, and depression

- The importance of lipids in the brain

Lipids and their metabolic intermediates are core components of brain structure and function, accounting for approximately 50% of the brain's dry weight (54). The fatty acid composition of the brain is unique, being rich in long-chain polyunsaturated fatty acids (LC-PUFAs), especially arachidonic acid (AA), EPA, and DHA (55). Dietary fatty acid intake can affect the fatty acid composition of different brain regions, thereby influencing mood and behavior (56–58).

- Direct association between omega-3 polyunsaturated fatty acids and depression

Numerous studies have confirmed that omega-3 PUFA deficiency (especially EPA and DHA) may induce depression (59–61). Deficiency is typically defined as serum EPA + DHA accounting for < 3% of total fatty acids (TFA)—a threshold validated in clinical studies linking low omega-3 status to higher depressive symptom severity (62, 63). As a lipid component abundant in the brain (64), Omega-3 polyunsaturated fatty acids are closely related to depression intervention:

**Clinical evidence:** patients with depression have lower levels of Omega-3 polyunsaturated fatty acids (62); a meta-analysis covering 26 studies showed that supplementation with EPA (≥1,000 mg/day) + DHA (≥200 mg/day) can improve depressive symptoms (65). For food sources: consuming 100–150 g of deep-sea fish (e.g., salmon, mackerel; containing ~2,000 mg EPA + 500 mg DHA per 100 g) daily meets the mood-beneficial intake (5, 66).

- Functional differences among different Omega-3 components:

**DHA:** DHA's high concentration in the frontal cortex is critical for maintaining the structure and function of neuronal membranes (e.g., lipid rafts) and neurotransmitter receptors (e.g., 5-HT2A). A reduction in frontal cortex DHA (not complete absence) impairs 5-HT signal transmission and neuroplasticity—key factors contributing to depressive symptoms, though not the sole cause of depression (67, 68). Supplementation with 200–500 mg/day of DHA (as adjuvant therapy) can improve mild to moderate depression (66);

**EPA:** Constitutes less than 1% of total fatty acids in the brain, but when supplemented at 1,000–2,000 mg/day (combined with 200–500 mg/day DHA), it can inhibit the reduction of neurogenesis and decrease the secretion of inflammatory factors (69) [these cytokines can induce apoptosis and neuroinflammation, and are positively correlated with depressive symptoms (70)];

**ALA:** as a precursor of Omega-3 fatty acids, it can be converted into DHA and EPA [the human body cannot synthesize it on its own and must obtain it through foods such as deep-sea fish (52)]. A longitudinal study showed that increased ALA intake can alleviate depressive symptoms (71), but its antidepressant effect remains controversial (62).

**Serum metabolic association:** serum levels of EPA and DHA are negatively correlated with moderate to severe depression (63, 72), further confirming the importance of dietary Omega-3 supplementation.

### 4.3 Potential mechanisms of omega-3 polyunsaturated fatty acids affecting depression

**(1) Regulating neurotransmitter transmission**

- Neuron membrane fluidity: DHA, as a major component of neuronal membrane phosphatidylcholine, modulates the structure of lipid rafts—specialized membrane microdomains that concentrate neurotransmitter receptors. Increased DHA incorporation into lipid rafts enhances the surface expression and dimerization of 5-HT2A receptors, improving their affinity for 5-HT (73, 74). Additionally, Omega-3 fatty acids inhibit phospholipase A2 (PLA2) activity, reducing the release of arachidonic acid (AA) from membranes. This limits the synthesis of pro-inflammatory prostaglandins [e.g., ProstaglandinE2 (PGE2)], which otherwise impair dopamine and 5-HT reuptake transporters (DAT and SERT) (75, 76).
- Interaction with the serotonin (5-HT) system: increased binding of DHA to cell membranes can enhance 5-HT sensitivity; the binding of 5-HT to the 5HT2A receptor can also mobilize the supply of DHA to neurons (73); the levels of EPA and DHA can affect the content and function of 5-HT in the brain (74, 77);
- Interaction with the dopamine system: both 5-HT and dopamine metabolism are regulated by Omega-3 fatty acids (78, 79);
- Core role: supplementation with Omega-3 fatty acids can exert antidepressant effects by enhancing neurotransmitter transmission (76).

**(2) Modulating immune and inflammatory responses**

Depression is often accompanied by an excessive inflammatory response of the immune system, characterized by increased levels of pro-inflammatory cytokines and linoleic acid metabolites (80–82). EPA and DHA are natural anti-inflammatory substances (83), and their anti-inflammatory mechanisms include:

- EPA and DHA compete with AA for cyclooxygenase (COX) and lipoxygenase (LOX) enzymes, leading to the production of specialized pro-resolving mediators (SPMs) such as resolvin E1 (RvE1) and protectin D1 (PD1) instead of pro-inflammatory leukotrienes (83, 84). RvE1 activates the GPR32 receptor on microglia, inhibiting the NF-κB pathway—this reduces the transcription of pro-inflammatory cytokines [IL-1β (Interleukin - 1β), IL-6, TNF-α) and suppresses neuroinflammation (85, 86). Moreover, EPA/DHA improve the Omega-6/Omega-3 ratio, and a lower ratio reduces the activation of toll-like receptor 4 (TLR4) on immune cells, further attenuating inflammatory signaling (86, 87).
- Reducing arachidonic acid metabolism and lowering pro-inflammatory products (prostaglandins, leukotrienes) (84, 85);
- Improving the Omega-6/Omega-3 fatty acid ratio: a higher ratio is associated with higher levels of pro-inflammatory cytokines (86, 87).

Therefore, Omega-3 fatty acids may achieve antidepressant effects primarily by regulating the functions of immune cells and pro-inflammatory cells.

### 4.4 The mediating role of gut microbiota between lipid metabolism and depression

The intestine is the main site of lipid metabolism. Gut microbiota can affect mood indirectly by transforming and synthesizing lipids, decomposing dietary lipids to produce regulatory metabolites (88–90). The specific pathways are as follows:

**(1) Association between gut microbiota and lipid metabolites**

Some gut microbiota (such as *Bacteroides, Clostridium, Lactobacillus, Bifidobacterium*, and *Ruminococcaceae*) are associated with lipid metabolites in the blood (89, 91–94). Depression can disrupt the structure of gut microbiota (45), and microbial metabolites [triglycerides, low-density lipoproteins, high-density lipoproteins, phosphatidylcholine, etc. (89, 91–94)] can affect human lipid metabolism (94–96), thereby influencing mood and cognitive function (97–99).

**(2) How do lipids influence SCFAs production?**

SCFAs (acetate, propionate, butyrate, etc.) are the end products of dietary fiber fermentation by gut microbiota, mainly synthesized by genera such as *Akkermansia, Bifidobacterium*, and *Faecalibacterium* (100–102). Lipids (especially omega-3 PUFAs) do not directly participate in carbohydrate fermentation but indirectly promote SCFA production by:

- Enhancing the abundance of SCFA-producing bacteria (e.g., Akkermansia, Roseburia) (88, 103)—these bacteria rely on omega-3 PUFAs to maintain cell membrane integrity and metabolic activity;
- Improving gut barrier function (via omega-3-mediated tight junction upregulation), reducing LPS-induced damage to SCFA-producing bacteria (44, 90);
- Modulating gut pH (via omega-3 metabolites), creating a favorable environment for fiber-fermenting bacteria (89).

Their functions include: maintaining intestinal barrier function (44), participating in serotonin synthesis (19), and regulating lipid metabolism (104, 105).

**Association with depression:** patients with depression have lower levels of acetate and propionate, and higher levels of isocaproic acid in their feces (24); Valeric acid, produced mainly by Oscillibacter, has a structure similar to GABA and can bind to its receptors (106). The content of *Oscillibacter* is higher in the feces of depressed patients (107), which may play an important role in severe depressive disorders.

**(3) There exists a distinct “dose-effect window” for the antidepressant effects of Omega-3, which should be stratified based on the severity of depression:**

- Mild depression: daily supplementation with 0.5–1.0 g of EPA combined with 0.2–0.5 g of DHA has been shown to alleviate symptoms. A meta-analysis (65) demonstrated that the BDI-II score in this dosage group decreased by 3.0 points (95% CI: −4.2 to −1.8), with no significant adverse effects reported.
- Moderate depression: a daily dose of at least 1.0 g of EPA, preferably combined with 0.5 g of DHA, is recommended. A 12-week randomized controlled trial (66) revealed that the depression remission rate in the group receiving 2.0 g of EPA and 0.5 g of DHA reached 58%, significantly higher than that in the low-dose group (32%, *P* < 0.05).
- Severe depression: omega-3 should be used in conjunction with antidepressant medications. The recommended daily dose of EPA is 1.5–2.0 g. Excessive intake (≥3.0 g/day) may increase the risk of gastrointestinal discomfort, such as diarrhea, without providing additional antidepressant benefits (65). Furthermore, the EPA/DHA ratio should be maintained at ≥2:1. A lower ratio (e.g., 1:1) may diminish the anti-inflammatory effects and consequently reduce the antidepressant efficacy (84).
- ALA supplementation for benefits: ALA has low conversion efficiency to EPA/DHA [ < 10% in humans (52)], so supplementing 2,000–3,000 mg/day of ALA may modestly alleviate depressive symptoms [only validated in longitudinal studies (71)], but it is less effective than direct EPA/DHA supplementation.

**(4) Summary and prospects**

Existing studies indicate that lipid metabolism is closely related to mood. It is hypothesized that lipids may affect depression through three main pathways:

- Omega-3 fatty acids act through the serotonin neurotransmitter system;
- Omega-3 fatty acids regulate the functions of immune cells and pro-inflammatory cells;
- Gut microbiota regulates lipid metabolism by influencing the levels of metabolic SCFAs, thereby indirectly affecting mood.

However, there is still insufficient evidence to explain the specific associations between lipid metabolism, gut microbiota, and mood, which remains a hot topic in current research.

### 4.5 Association between omega-3 fatty acids intake and depression, and research limitations

The cited evidence has gaps, including overreliance on observational data and heterogeneous intervention studies:

- First, mechanistic research is dominated by animal studies—the link between omega-3s and neurogenesis/inflammation (69) relies on mouse models of chronic stress, yet human depression involves complex cognitive and social factors, and the fatty acid composition of the mouse brain (e.g., DHA accounts for ~30 vs. 40% in humans (67)) differs, which limits the extrapolation of such findings (69, 70);
- Second, intervention studies exhibit high heterogeneity—the cited meta-analysis (65) includes 26 studies with variable doses (EPA/DHA ranges: 0.2–3 g/day) and varying degrees of depression severity (from mild to severe), and subgroup analysis shows that only high-dose EPA (>1 g/day) has a small effect on moderate depression, while low-dose supplements (≤ 0.5 g/day) provide no benefit—this dose-response relationship was not discussed in the original analysis (65, 66);
- Third, observational studies overstate correlations—serum EPA/DHA levels (63, 72) may be a marker of an overall healthy diet (e.g., high fish intake often coincides with high fiber and vitamin intake) rather than a direct driver of depression (62, 72).
- Fourth, regarding the “microbiota-lipid-depression” mediation hypothesis, human intervention data are scarce—most evidence [e.g., (88–90) on lipid transformation] comes from germ-free mouse models, but the diversity of the human gut microbiota (e.g., ~1,000 species vs. ~200 in mice) and lipid metabolism pathways (e.g., bile acid synthesis) are different, rendering animal study results non-translatable to humans (88–90);
- Fifth, confounding factors remain unaddressed—dietary fiber (a major precursor of SCFAs) is often consumed alongside omega-3s (e.g., in fatty fish), so changes in SCFAs (24) may be driven by fiber rather than omega-3s, a confounder that was ignored in the original analysis (24, 102).

## 5 Sugars (dietary fiber), the gut microbiota, and depression

Carbohydrates serve as the primary energy source for all living organisms to sustain life activities. With the improvement of living standards, sugar-containing foods—especially children's foods and sugary beverages—are ubiquitous. A series of studies have demonstrated that excessive consumption of high-sugar foods disrupts the body's normal glucose metabolism, leading to metabolic diseases such as diabetes, hypertension, and obesity (108–110). One study revealed that a high-sugar diet is associated with an increased risk of 45 diseases, including 18 endocrine disorders, 10 cardiovascular diseases, seven types of cancer, and 10 other conditions (depression included) (111). However, dietary carbohydrates encompass not only monosaccharides (e.g., glucose, fructose) and disaccharides (e.g., sucrose, lactose) but also polysaccharides and added sugars (artificial sweeteners) (112). This article focuses on dietary fiber (a type of polysaccharide) and explores its correlative mechanisms with gut microbiota and depression.

### 5.1 Classification and main sources of dietary fiber

Dietary fiber is a class of carbohydrates found in plant-based foods such as whole grains, vegetables, fruits, and legumes (113). Based on the physiological properties of monomer unit (MU) polymerization, it can be categorized into three main types:

- **Non-starch polysaccharides (NSPs):** with a monomer unit count (MU) ≥10. Inulin is a common example, primarily derived from foods like onions, garlic, and bananas;
- **Resistant starch (RS):** with a monomer unit count (MU) ≥10. It can be further divided into RS1 to RS5 based on sources and characteristics, such as milled grains and seeds (RS1), raw potatoes/corn/unripe bananas (RS2), cooked and cooled potatoes and cornflakes (RS3), baked products (RS4), and fried rice flakes (RS5) (114);
- **Resistant/indigestible oligosaccharides (RIOS):** with a monomer unit count (MU) of 3–9. Examples include fructooligosaccharides and β-glucan [the most physiologically active type of glucan, known as “immune gold,” widely present in plants and fungi like oats and mushrooms (115)].

### 5.2 Interaction between dietary fiber and gut microbiota

The human body cannot secrete the polysaccharide hydrolases required to decompose dietary fiber independently. However, gut microbiota can produce a variety of polysaccharide hydrolases to degrade dietary fiber and utilize it as an energy source (116). Different types of dietary fiber exhibit specific regulatory effects on the composition of gut microbiota:

**(1) Regulation of gut microbiota by NSPs**

Inulin, a representative NSP, can significantly increase the abundance of beneficial bacteria in the gut. Studies have shown that inulin supplementation increases the abundance of *Bifidobacterium* by 8.38%, Lactobacillus by 0.26%−1.26%, and *Faecalibacterium* by 0.2% (117), supporting the balance of the intestinal microecosystem.

**(2) Regulation of gut microbiota by RS**

When the diet is rich in resistant starch, the abundance of specific gut bacteria changes: the counts of *Faecalibacterium, Roseobacter*, and *Ruminococcus* increase significantly (114). The proliferation of these bacteria helps enhance intestinal metabolic function, laying the foundation for subsequent metabolite production.

**(3) Regulation of gut microbiota by RIOS**

Different types of oligosaccharides exert targeted regulation on gut microbiota:

- **Fructooligosaccharides:** promote the increased abundance of *Bifidobacterium, Lactobacillus, Faecalibacterium, Ruminococcus, Sutterella*, and *Oscillospira* (118);
- **β-glucan:** animal experiments have shown that oat β-glucan can increase the abundance of *Bifidobacterium* and *Lactobacillus* in the intestines of rats (119, 120). A clinical study revealed that adding 3 g/day of high-molecular-weight (HMW) β-glucan to the diet increases the abundance of Bacteroides and Prevotella while decreasing the abundance of Dorea (121).

Notably, previous studies have confirmed that the aforementioned gut bacteria regulated by dietary fiber (e.g., *Bifidobacterium, Lactobacillus, Faecalibacterium*) are closely associated with depression (45), providing a critical link for dietary fiber to intervene in depression via gut microbiota.

### 5.3 Potential mechanisms of dietary fiber affecting depression

**(1) Mood regulation mediated by SCFAs produced via gut microbiota fermentation**

**Figure 2:**
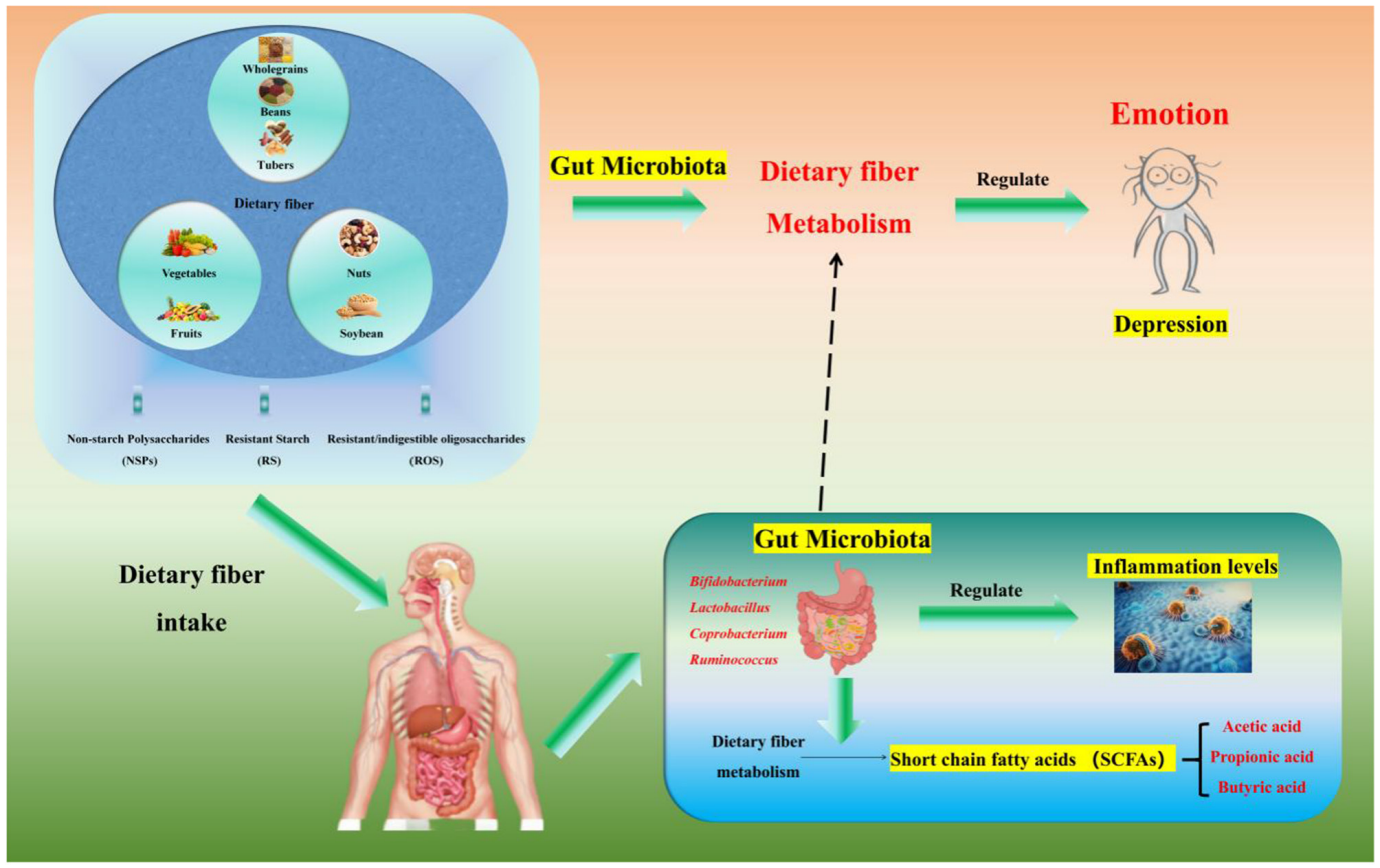
Relationships among dietary fiber, the intestinal microbiota and mood. Dietary fiber (a polysaccharide, including non-starch polysaccharides/NSPs such as inulin from onions, resistant starch/RS such as cooled potato starch, and resistant/indigestible oligosaccharides/RIOS such as fructooligosaccharides—each targeting specific gut bacteria) regulates gut microbiota and alleviates depression via metabolite short-chain fatty acids (SCFAs) mediation: it remodels gut microbiota by increasing *Bifidobacterium, Lactobacillus*, and *Faecalibacterium* abundance through NSPs/RIOS and enriching *Roseburia* and *Ruminococcus* (key butyrate-producing bacteria) through RS, and exerts anti-depressant effects via SCFAs [acetate/propionate activate GPR41/43 to upregulate tight junction proteins (ZO-1, Occludin) for intestinal barrier protection by reducing LPS translocation; butyrate crosses the blood-brain barrier via MCT1 to inhibit HDACs and promote BDNF expression for neuroplasticity promotion, which is critical for neuronal survival; SCFAs suppress the NLRP3 inflammasome to reduce intestinal and systemic inflammation, breaking the “inflammation-depression” cycle], with depressed patients typically showing reduced fecal acetate/propionate levels that align with the fiber-microbiota-SCFA-mood axis.

Dietary fiber cannot be digested and absorbed by the human body but can be partially or fully fermented by gut microbiota (122). This fermentation process generates various byproducts, among which SCFAs are the core pathway connecting gut microbiota and host metabolic interactions (consistent with the previously mentioned mechanism by which proteins and lipids improve mood through the microbiota-SCFAs axis). The specific functional logic is as follows:

- **In addition to GPR activation, SCFAs (especially butyrate) cross the blood-brain barrier via monocarboxylate transporter 1 (MCT1) and inhibit HDAC1/2 in the hippocampus and prefrontal cortex—brain regions critical for mood regulation**. HDAC inhibition increases acetylation of the NR3C1 gene (encoding the glucocorticoid receptor), enhancing glucocorticoid receptor sensitivity and restoring the negative feedback of the hypothalamic-pituitary-adrenal (HPA) axis. This reduces chronic stress-induced cortisol overproduction, a key driver of depressive symptoms (19, 105, 123). Furthermore, propionic acid stimulates enteric neurons to release glutamate, which activates vagal afferents to transmit signals to the CNS, modulating mood-related brain circuits (19, 20).
- **Foundation for SCFA production:** dietary fiber (especially fructans and galactooligosaccharides) can significantly increase the abundance of *Bifidobacterium* and *Lactobacillus* in the gut (124), and these two bacterial groups are the main producers of SCFAs (100–102);
- **Mood-regulating effect of SCFAs:** Studies have confirmed that *Bifidobacterium* and *Lactobacillus* can improve depression-like behaviors (20, 125, 126). For example, a study by Pinto-Sanchez et al. showed that supplementation with Bifidobacterium longum NCC3001 (BL) at a dose of 1 × 10^10^ CFU/day for 4 weeks improves depression-like symptoms (reduced BDI-II score by 3.2 points) and quality of life (increased IBS-QoL score by 15%) in IBS patients with comorbid depressive symptoms (20).
- **Summary of core mechanisms:** dietary fiber → increased abundance of gut microbiota (*Bifidobacterium*/*Lactobacillus*) → enhanced SCFA production → mood regulation via the “gut-brain axis” → alleviation of depressive symptoms (Figure 2).

**(2) Improvement of depression by regulating inflammatory levels**

Inflammation is closely associated with mood, as evidenced by the following: patients with acute symptomatic psychosis often exhibit acute inflammatory states; psychological stress—a major risk factor for depression—can induce inflammatory responses and increase inflammatory markers in healthy volunteers (127–129); cytokines are involved in the development of depressive symptoms, and environmental factors may also trigger both depression and immune disorders (127–129). Additionally, the levels of anti-inflammatory cytokines are often elevated in patients with major depressive disorder (130, 131), further highlighting the key role of inflammation in depression.

Dietary fiber-derived SCFAs suppress intestinal inflammation by inhibiting the activation of the NLRP3 (NLR family pyrin domain containing 3) inflammasome—a multiprotein complex that mediates caspase-1-dependent maturation of IL-1β. GPR43 activation by SCFAs reduces intracellular ATP depletion and reactive oxygen species (ROS) production, which are critical for NLRP3 assembly (132, 133). Furthermore, fiber fermentation increases gut *Faecalibacterium prausnitzii* abundance; this bacterium produces anti-inflammatory metabolites (e.g., butyrate androsmarinic acid) that downregulate the expression of TLR4 and CD14 (Cluster of Differentiation 14) on intestinal macrophages, limiting LPS (Lipopolysaccharides)-induced inflammatory responses (132, 134).

Dietary fiber can indirectly intervene in depression by regulating inflammatory levels, supported by the following research evidence:

- A prospective cohort study involving 4,125 elderly individuals showed that higher cereal fiber intake is associated with lower levels of various inflammatory markers (132);
- A study by Wastyk et al. (133) indicated that dietary interventions rich in dietary fiber and fermented foods have the potential to increase gut microbiota diversity and reduce the levels of inflammatory markers (133).

Thus, dietary fiber can break the “inflammation-depression” vicious cycle by reducing systemic inflammatory levels, thereby improving depressive symptoms (Figure 2).

### 5.4 Different kinds of dietary fibers and their diverse effects on gut inflammation

Section 5.3 has partially discussed the regulatory role of dietary fiber in gut inflammation; however, the differential effects of various dietary fiber types on gut inflammation warrant further elaboration. Based on the existing evidence and the central framework of this review, the specific distinctions and underlying mechanisms are further detailed as follows (Table 1):

**Table 1:** The differences in gut inflammation regulation among major dietary fiber types.

**Dietary fiber type**	**Representative examples**	**Key microbiota targets**	**Anti-inflammatory mechanism**	**Evidence source**
NSPs	Inulin, oat β-glucan	*Bifidobacterium, Faecalibacterium*	Butyrate-mediated NF-κB inhibition; tight junction enhancement	(117, 121, 132)
RS	Cooled potato starch (RS3)	*Roseburia, F. prausnitzii*	NLRP3 inflammasome suppression; mucosal barrier repair	(114, 135)
ROS	Fructooligosaccharides (FOS)	*Lactobacillus, Bifidobacterium*	GPR43 activation; antimicrobial peptide secretion	(118, 122)

**(1) NSPs: targeted inhibition of pro-inflammatory pathways**

As one of the most extensively studied categories of dietary fiber (see Section 5.1), NSPs, such as inulin and β-glucan, demonstrate significant anti-inflammatory properties through modulation of gut microbiota and enhancement of SCFAs production.

- **Inulin:** clinical studies indicate that inulin supplementation at a dosage of 10–15 g/day leads to an 8.38% increase in *Bifidobacterium* and a 0.2% increase in *Faecalibacterium* in the human gut (117). These bacteria ferment inulin into butyrate, which inhibits the phosphorylation of Nuclear Factor kappa-B (NF-κB) p65 in colonic epithelial cells, thereby reducing the secretion of pro-inflammatory cytokines such as IL-6 and TNF-α (44, 132). In a RCT involving 120 patients with mild gut inflammation, an 8-week inulin intervention resulted in a 32.6% reduction in fecal calprotectin, a recognized biomarker of gut inflammation (124).
- **β-glucan:** high-molecular-weight β-glucan derived from oats (3 g/day) has been shown to increase the abundance of *Bacteroides* and *Prevotella* while decreasing pro-inflammatory Dorea species (121). Experimental studies in animal models have confirmed that oat β-glucan enhances the expression of intestinal tight junction proteins, including Occludin and ZO-1, thereby improving gut barrier integrity and reducing LPS-induced systemic inflammation (119, 120).

**(2) RS: Regulation of inflammation via microbiota-metabolite axis**

RS, such as RS3 derived from cooled potatoes, differs from NSPs in terms of fermentation rate and anti-inflammatory targets, demonstrating particularly pronounced effects on colonic mucosal inflammation.

- **Microbiota remodeling:** a diet rich in resistant starch has been shown to increase the abundance of *Faecalibacterium* and *Roseburia*—key butyrate-producing bacteria—by 2.1–3.5 times in the human gut (114). *Faecalibacterium prausnitzii*, a representative RS-responsive bacterium, produces butyrate and anti-inflammatory peptides that inhibit the activation of the NLRP3 inflammasome in colonic macrophages (135).
- **Clinical evidence:** in a cohort study involving 4,125 elderly individuals, higher cereal-derived RS intake (≥15 g/day) was associated with a 28% reduction in serum IL-6 levels and a 35% reduction in TNF-α levels compared to low-RS intake (< 5 g/day) (132). This effect is primarily attributed to RS-derived butyrate, which enhances intestinal barrier function and reduces LPS translocation (44).

**(3) RIOS: modulation of local inflammatory microenvironment**

RIOS, such as FOS (Fructo - OligoSaccharide), are characterized by shorter monomer chains (degree of polymerization 3–9) and rapid fermentation, making them particularly effective in alleviating mild-to-moderate gut inflammation.

- **FOS:** FOS supplementation at a dosage of 5–8 g/day promotes the proliferation of *Bifidobacterium* and *Lactobacillus*. The metabolite acetate produced by these bacteria inhibits the migration of neutrophils to the colonic mucosa (118). In a murine model of dextran sulfate sodium (DSS)-induced colitis, FOS intervention reduced the colonic mucosal damage score by 40% and downregulated the expression of pro-inflammatory genes, including IL-1β and iNOS (122).
- **Mechanistic specificity:** unlike NSPs, which primarily modulate the NF-κB signaling pathway, RIOS exert their anti-inflammatory effects through activation of G protein-coupled receptor 43 (GPR43) on intestinal epithelial cells. This activation enhances the secretion of antimicrobial peptides, such as defensins, and suppresses the colonization of pro-inflammatory bacterial species, including Enterobacteriaceae (19, 100).

### 5.5 Association between dietary fiber intake and depression, and research limitations

The SCFA-mediated pathway has notable limitations, including overreliance on animal models and unproven causality.

- First, animal studies overshadow human mechanistic data: the link between SCFAs and intestinal 5-HT synthesis (19) relies on mouse ECs experiments, but human ECs produce ~90% of body 5-HT vs. ~70% in mice, and SCFA receptors (e.g., GPR41) have different expression patterns in human vs. mouse brains (19, 123).
- Second, observational studies have methodological flaws: the cited study (24) (depressed patients have lower acetate/propionate) is small (*n* = 30) and does not control for dietary fiber intake—depressed patients often consume less fiber, so SCFA changes may be a result of fiber deficiency rather than a direct driver of depression (24, 124).
- Finally, mediation is not confirmed: the original analysis assumes SCFAs “mediate” fiber-mood effects, but no RCT has directly validated that increasing SCFAs (e.g., via butyrate supplements) alleviates depressive symptoms in humans (20, 102).

## 6 Vitamins, gut microbiota, and depression

Vitamins are organic compounds essential for maintaining human health. As regulatory substances, they play a crucial role in material metabolism. However, the human body cannot synthesize these substances or produces them in insufficient quantities, so they must be primarily obtained from food. Vitamins are generally classified into fat-soluble vitamins and water-soluble vitamins:

- **Fat-soluble vitamins** are metabolized in the body similarly to fats and serve as components of cell membranes.
- **Water-soluble vitamins** mostly act as coenzymes in metabolic reactions, carrying chemical groups and electrons, and exhibit specific physiological functions (136, 137).

For instance, certain vitamins (e.g., vitamin A and vitamin C) possess direct antibacterial effects *in vitro* or *in vivo* (138, 139). Additionally, water-soluble vitamins diffuse through the intestinal wall into the bloodstream, while fat-soluble vitamins are emulsified and encapsulated in lipid-rich micellar mixtures containing fatty acids, bile salts, and phospholipids. These fat-soluble vitamins then pass through the brush border (villi), are absorbed into the lymphatic circulation, and ultimately delivered to tissues, target cells, or organs (137).

Recent studies have shown that the gut microbiota also functions as a “producer” of vitamins, contributing to the adequacy of micronutrients and the stability of gut microbial communities (140). Dysbiosis of the gut microbiota and vitamin deficiency are interconnected, and this relationship may directly affect host health—vitamin intake alters the composition and biological functions of the gut microbiota (141–143). Although vitamins are not used as energy sources, they can interact bidirectionally with the gut microbiota through direct or indirect means. Furthermore, growing evidence indicates that nutritional regulation of the gut microbiota is a potentially beneficial therapeutic strategy.

### 6.1 Water-soluble vitamins: classification, interaction with gut microbiota, and mechanisms

Water-soluble vitamins mainly include B-group vitamins (B1, B2, B3, B5, B6, B7, B9, and B12) and vitamin C. Among them, B-group vitamins can be synthesized by the gut microbiota (144), while vitamin C can be synthesized by the gut microbiota in addition to dietary supplementation (145).

**(1) Effects of B-group vitamins on the gut microbiota**

B-group vitamins maintain the balance of the intestinal microecosystem by regulating the abundance of specific microbiota, supported by the following research evidence:

- **Regulation of microbiota abundance:** studies have shown that vitamin B2 can increase the abundance of *Alistipes* and *Clostridium* (146); Carrothers et al. (147) found that increased intake of vitamins B2, B5, B6, and B12 was associated with higher relative abundance of *Prevotella* and lower relative abundance of *Bacteroides* in fecal samples (147);
- **Capacity of microbiota to synthesize B-group vitamins:** Magnúsdóttir et al. identified B-group vitamin biosynthetic pathways in 256 common gut bacteria through systematic genomic analysis. They found that the human gut microbiota can synthesize eight out of nine B-group vitamins (except vitamin B12). *Bacteroidetes, Firmicutes*, and *Proteobacteria* are closely associated with B-group vitamin synthesis. Specifically, the gut microbiota can provide 3% of the Recommended Daily Allowance (RDA) for vitamin B2, 27% for vitamin B3, 86% for vitamin B6, 37% for vitamin B9, and 31% for vitamin B12 (144), confirming a synergistic relationship between the gut microbiota and B-group vitamins; vitamin B6 (pyridoxal 5'-phosphate, PLP) acts a coenzyme for kynurenine transaminase (KAT) in the tryptophan-kynurenine pathway (KP). Adequate B6 availability shifts KP metabolism toward the production of nicotinamide adenine dinucleotide (NAD+) instead of neurotoxic quinolinic acid (QUIN)—QUIN activates N-methyl-D-aspartate (NMDA) receptors excessively, leading to neuronal excitotoxicity and depression (144, 148). Additionally, vitamin B12 (cobalamin) is required for methionine synthase activity, which converts homocysteine to methionine. Low B12 levels increase homocysteine, which impairs methylation of DNA and proteins (e.g., BDNF) and enhances oxidative stress—both linked to depressive phenotypes (147, 149).
- **Transport and utilization of B-group vitamins by microbiota:** Rodionov et al. conducted computational simulations of B-group vitamin biosynthesis, salvage, and uptake in 2,228 bacterial genomes representing 690 human gastrointestinal microbiota. They confirmed that the human gastrointestinal microbiota can provide transporters for vitamins and their precursors (149), further strengthening the association between vitamins and the gut microbiota;
- **Support of B-group vitamins for butyrate-producing bacteria:** Soto-Martin et al. (148) investigated the requirements of 15 strains of human gut butyrate-producing bacteria for eight B-group vitamins and proteinogenic amino acids through a combination of genomic sequence analysis and *in vitro* growth experiments. The results showed that B-group vitamins can support the growth of two Ruminococcaceae species (*F. prausnitzii* and *S. variabile*), indicating that some butyrate-producing bacteria depend on dietary B-group vitamins (148).

**(2) Effects of vitamin C on the gut microbiota**

Vitamin C (ascorbic acid) has attracted considerable attention due to its well-documented antioxidant and anti-inflammatory properties (150, 151). However, research on the relationship between vitamin C and the gut microbiota remains limited, with only two clinical trials exploring the effects of vitamin C supplementation on the gut microbiota:

- Otten et al. showed that supplementing healthy individuals with high-dose vitamin C increased the abundance of *Lachnospiraceae*, but decreased the abundance of *Bacteroidetes, Enterococci*, and *Gemmiger formicilis* (152);
- Subsequent research by Hazan et al. found that vitamin C increased the levels of Bifidobacterium in the gut (153).

### 6.2 Fat-soluble vitamins: classification, interaction with gut microbiota, and mechanisms

Fat-soluble vitamins mainly include vitamin A, vitamin D, vitamin E, and vitamin K (137). Among them, vitamin A, vitamin D, and vitamin E are primarily obtained through dietary supplementation and absorbed via metabolism in the small intestine (154–156); in addition to dietary intake, vitamin K can also be synthesized by the gut microbiota (157, 158).

**(1) Effects of vitamin A on the gut microbiota**

Vitamin A improves vision, regulates growth and development, and modulates immune function. It is mainly derived from retinol in meat and fish, and carotenoids in fruits and vegetables (159). Since 70%−90% of vitamin A is absorbed in the gut (160), it has a potential association with the gut microbiota, as evidenced by:

- **Clinical research evidence:** Vitamin A supplementation promotes the growth of *Bifidobacteria, Actinobacteria, Proteobacteria, Akkermansia*, and *Clostridia*; in contrast, vitamin A deficiency increases the abundance of *Enterococcus* (161– 163);
- **Animal experiment evidence:** Some animal model studies found that vitamin A can regulate the abundance of *Lactobacillus* and *Clostridium* (164, 165);
- **Metabolic association:** The aforementioned microbiota regulated by vitamin A are all associated with SCFAs and tryptophan metabolism (166, 167), providing clues for vitamin A to affect host metabolism through microbiota.

**(2) Effects of vitamin D on the gut microbiota**

Vitamin D deficiency is associated with intestinal diseases such as ulcerative colitis, Crohn's disease (CD), and other inflammatory bowel diseases (168, 169). Based on studies of these intestinal diseases, vitamin D has been confirmed to regulate the growth of the gut microbiota, with specific findings as follows:

Vitamin D exerts its effects by binding to the vitamin D receptor (VDR), a nuclear transcription factor. In intestinal epithelial cells, VDR forms a heterodimer with the retinoic acid X receptor (RXR) and binds to vitamin D response elements (VDREs) in the promoter regions of genes encoding antimicrobial peptides (AMPs), such as defensins and cathelicidins (103, 170). AMPs selectively inhibit the growth of pro-inflammatory bacteria (e.g., *Enterobacteriaceae*) while promoting the proliferation of beneficial taxa (e.g., *Roseburia, Akkermansia)* (170, 171). Additionally, VDR activation upregulates tight junction proteins (ZO-1, occludin) and downregulates pro-inflammatory cytokines (IL-6, TNF-α) by inhibiting NF-κB, thereby linking gut microbiota balance to reduced systemic inflammation and depression (103, 172).

**Study on CD patients:** Schäffler et al. (103) conducted oral vitamin D intervention in CD patients and found that 1 week after vitamin D1 supplementation, the abundances of Alistipes, Barnesiella, unclassified Porphyromonadaceae, Roseburia, Anaerotruncus, Subdoligranulum, and unclassified Ruminococcaceae in the patients' guts increased significantly (103);

**Study on mouse colitis model:** Ooi et al. (171) found that vitamin D regulated the composition of the gut microbiota (including Bacteroidetes, Proteobacteria, Firmicutes, Deferribacteres, Lactobacillaceae, and Lachnospiraceae) in a mouse colitis model induced by dextran sulfate sodium (171);

**Mother-infant cohort studies:** two large-scale cohort studies on the effects of vitamin D supplementation on the infant gut microbiota showed that maternal diet and plasma vitamin D levels were negatively correlated with Bifidobacterium and Clostridioides difficile in infants (173); moreover, maternal vitamin D supplementation may reduce the growth of Clostridioides difficile in infants (172).

Although the above studies have specific designs, they all confirm that vitamin D has the ability to regulate the gut microbiota (170).

**(3) Effects of vitamin E on the gut microbiota**

The natural sources of vitamin E are mainly the oily components of nuts and oilseeds, which exhibit antioxidant, anti-inflammatory, anti-aging, and anti-cancer properties (174, 175). Vitamin E also interacts with the gut microbiota, supported by the following evidence:

**Study on maternal gut microbiota:** a study exploring the relationship between dietary intake and maternal gut microbiota showed that higher vitamin E intake was associated with lower levels of *Proteobacteria* (especially Sutterella) (176). *Proteobacteria* have pro-inflammatory properties, and *Sutterella* is highly abundant in the guts of autistic patients (177);

**Fe (Iron) and vitamin E supplementation trial in infants:** Tang et al. (178) conducted a randomized trial of iron and vitamin E supplementation in Fe-deficient infants in the United States. They found that higher serum vitamin E concentrations in infants were associated with higher relative abundance of *Roseburia* (a butyrate-producing bacterium) (178);

***In vitro*** **and animal experiments:** Pham et al. found that vitamin E increased the relative abundances of *Akkermansia, Bifidobacterium*, and *Faecalibacterium*, while also increasing the levels of acetate, butyrate, and propionate (146); supplementation with tocotrienols (one of the main natural forms of vitamin E) increased the level of *Verrucomicrobia* in the guts of mice (179).

Currently, research on the effects of vitamin E on the gut microbiota remains limited, lacking systematic study validation.

**(4) Effects of vitamin K on the gut microbiota**

Vitamin K is mainly obtained from green leafy vegetables and vegetable oils in the diet. It can also be acquired from menadione in fermented foods or through biosynthesis by the gut microbiota (158). Its main function is anticoagulation (180), and it also regulates osteocalcin synthesis (181), inhibits inflammation (182), and suppresses the growth of certain cancer cells (183, 184). Research on vitamin K and the gut microbiota is scarce, with existing evidence as follows:

**Study on CD patients:** Wagatsuma et al. (185) explored the relationship between the gut microbiota and vitamin K deficiency in CD patients and found that vitamin K deficiency significantly reduced the diversity of the gut microbiota, including *Ruminococcaceae* and *Lachnospiraceae*;

**Study on diet and microbiota in Japanese population:** Seura et al. (186) investigated the relationship between habitual dietary intake and the gut microbiota in the Japanese population. They found that young Japanese women with high vitamin K intake had higher relative abundances of *Bifidobacterium* and *Lactobacillales* in their guts (186);

**Study on fermented foods and menadione:** A study exploring the effects of a diet high in whole or refined grains on *in vivo* (fecal/serum) menadione concentrations and gut microbiota composition in men and postmenopausal women showed that menadione increased the abundances of *Bacteroides* and *Prevotella* (187);

**Animal experiments:** In vitamin K-deficient female C57BL/6 mice, the abundances of *Lachnospiraceae* and *Ruminococcaceae* in the gut were reduced (188), consistent with the findings of Wagatsuma et al. However, gender differences may exist in the effects of vitamin K deficiency on the gut microbiota (189), leading to insufficient evidence to explain the relationship between vitamin K and the gut microbiota.

Furthermore, recent studies have found that the gut microbiota affects patients' responses to anticoagulants and the vitamin K antagonist warfarin (190, 191). Therefore, there is an urgent need to strengthen basic research on the relationship between vitamin K and the gut microbiota.

### 6.3 Potential mechanisms of vitamins regulating depression via the gut microbiota

Based on the above research on the relationship between vitamins and the gut microbiota, it can be concluded that both water-soluble vitamins (B-group vitamins, vitamin C) and fat-soluble vitamins (vitamin A, D, E, and K) can alter the composition of the gut microbiota. They can also promote the growth of probiotics such as *Bifidobacterium, Faecalibacterium, Akkermansia, Clostridia*, and *Lactobacillus* (192)—these microbiota have been confirmed to be negatively associated with depression (45). The specific mechanisms by which vitamins regulate depression can be summarized into two points:

**(1) Improving depression by promoting probiotic growth and SCFA metabolism**

- **Role of water-soluble vitamins:** studies by Hazan and Martin et al. found that B-group vitamins and vitamin C can promote the growth of *Bifidobacterium* and *Ruminococcus* (148, 153), and these two types of microbiota are the main producers of SCFAs (193–195). As previously confirmed, SCFAs are the core pathway linking the gut microbiota to emotional changes (123), and changes in SCFAs in depressed mice are directly related to changes in the gut microbiota (196);
- **Role of fat-soluble vitamins:** Studies by Tian and Schaffler et al. found that vitamins A, D, E, and K can increase the abundance of butyrate-related bacteria (*Akkermansia, Ruminococcus, Clostridium, Roseburia, Coprococcus*) (103, 146, 165, 188, 197). Butyrate deficiency has been confirmed to be associated with depressive symptoms (135, 198).

Thus, dietary vitamin supplementation can regulate and prevent depressive mood by promoting probiotic growth and optimizing SCFA metabolism.

**(2) Counteracting mood-related damage by enhancing anti-inflammatory capacity**

**Figure 3:**
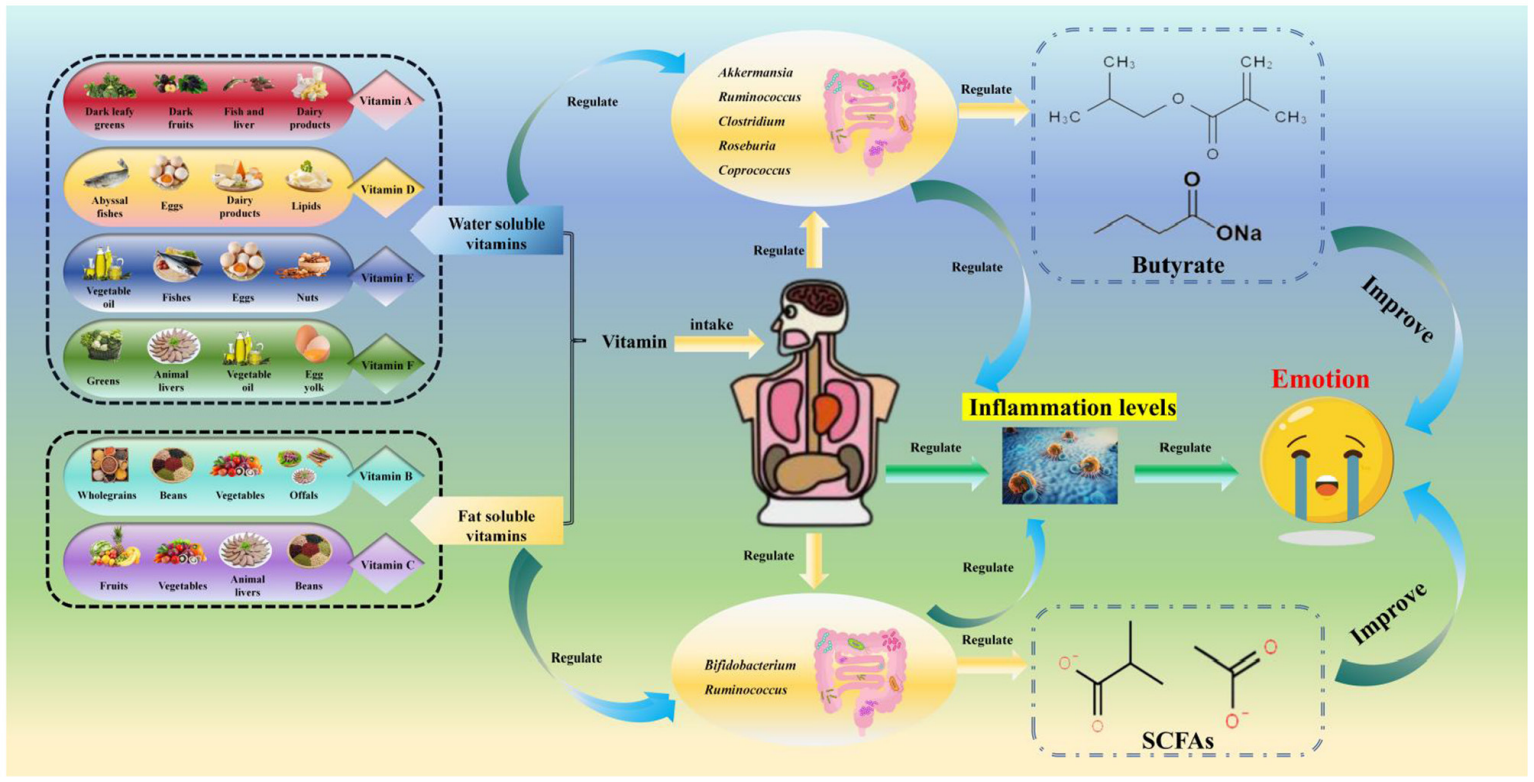
Relationships among vitamins, the gut microbiota, and mood. The categories, sources, gut microbiota-regulating effects, and anti-depressant mechanisms of vitamins: it first presents vitamin categories and their sources [fat-soluble vitamins: vitamin A from dark leafy greens/liver, vitamin D from deep-sea fish/dairy, vitamin E from nuts/vegetable oil, vitamin K from green leafy vegetables/gut microbiota synthesis; water-soluble vitamins: B-group vitamins (B6/B12) from whole grains/offals, vitamin C from fruits/vegetables], then details their gut microbiota regulation [vitamin D/A increase *Akkermansia, Roseburia*, and *Bifidobacterium* abundance, with vitamin D activating vitamin D receptors (VDR) to produce antimicrobial peptides and inhibit pro-inflammatory bacteria; B-group vitamins promote *Faecalibacterium* growth (supporting SCFA production) and are synthesized by gut microbiota such as B6/B12 by *Bacteroidetes*; vitamin C enriches *Lachnospiraceae* and *Bifidobacterium*, vitamin E increases *Roseburia*, while evidence for vitamin E/K remains limited], and finally explains their anti-depressant mechanisms (vitamins D/B6/B12 enhance SCFA production via beneficial bacteria to regulate the HPA axis and neuroplasticity; vitamin D/A reduce systemic inflammation by inhibiting NF-κB signaling; vitamin B6 acts as a coenzyme in the tryptophan-kynurenine pathway to reduce neurotoxic quinolinic acid production).

Negative emotions can increase *in vivo* inflammatory levels (21, 22), and vitamins can offset the damage to the host caused by negative emotions by enhancing the body's anti-inflammatory capacity (179, 199, 200), thereby indirectly improving depressive states (Figure 3).

### 6.4 Association between dietary fiber intake and depression, and research limitations

The claim of a “synergistic relationship” between B-group vitamins and the gut microbiota is overstated, as the supporting evidence has critical quality limitations.

- First, the evidence is heavily reliant on *in vitro* and animal studies: the proposed link between B-group vitamins and butyrate-producing bacteria (148) is derived from *in vitro* culture experiments, while germ-free mouse studies (149) fail to replicate key features of the human gut environment—such as physiological oxygen levels and inter-bacterial nutrient competition—undermining the translational relevance of their findings.
- Second, observational studies cited to support the relationship cannot establish causality: the study noting a correlation between B vitamin intake and Prevotella abundance (147) does not account for confounding factors—for instance, high B vitamin intake often coincides with increased vegetable consumption, a dietary factor that independently promotes Prevotella growth (147), making it impossible to attribute Prevotella changes solely to B vitamins.
- Finally, there is a lack of robust human intervention evidence: no RCTs have confirmed that B vitamin supplementation alters gut microbiota composition in a way that improves depression.

## 7 Mineral elements, gut microbiota, and depression

Mineral elements in the human body, also referred to as inorganic salts, are closely associated with human health. They participate in metabolic processes but cannot be produced or synthesized by the human body itself. Therefore, the host primarily acquires these nutrients through dietary supplementation (201, 202). Based on their effects on human health, mineral elements are generally categorized into essential elements, non-essential elements, and toxic elements (203). These elements have been shown to be involved in multiple physiological functions:

- **Structural functions:** constituting bone and soft tissues;
- **Regulatory functions:** mediating neuromuscular transmission, blood coagulation, oxygen transport, and enzyme activity (204–206);
- **Immunomodulatory activities** (207).

Deficiencies in mineral elements can lead to various diseases. For example, patients with neurodegenerative diseases often exhibit zinc (Zn) deficiency (208); calcium (Ca) deficiency may cause chronic conditions such as osteoporosis, arterial hypertension, and colon cancer (209, 210); and low Fe intake can result in iron deficiency anemia (211).

### 7.1 Inorganic salts and the gut microbiota

Mineral elements are essential for sustaining human life activities and normal physiological functions, and the gastrointestinal tract serves as the primary site for their absorption and metabolism. However, comprehensive studies on the relationship between the gut microbiota and mineral elements remain limited, with most research focusing on essential elements (205, 212–214). This section therefore discusses the associations between five key essential elements (Ca, Mg, Fe, Zn, and Se) and the gut microbiota.

**(1) Ca and the gut microbiota**

Ca is an essential element for the human body. As an enzyme activator, it participates in biological pathways such as bioelectrical impulse conduction, blood coagulation, muscle contraction, inflammation, and hormone secretion (215, 216). Dairy products are the primary dietary sources of Ca, with milk, yogurt, and cheese being the most common. After ingestion, Ca is mainly absorbed in the small and large intestines (217). Research on the Ca-gut microbiota relationship has primarily focused on animal studies related to osteoporosis:

- In a study where osteoporosis (induced by ovariectomy) in rats was ameliorated by modifying the gut microbiota, reducing the *Firmicutes*-to-*Bacteroidetes* ratio in the rat intestine promoted an increase in blood Ca ion concentration (218);
- Supplementation with *Lactobacillus acidophilus* and *Lactobacillus casei* improved Ca absorption in osteoporotic rats (219);
- Dietary Ca, acting in a prebiotic-like manner, significantly increased the abundances of *Bacteroidetes, Actinobacteria, Prevotella*, and *Bifidobacteria* in the gut of obese rats (220).

While the specific mechanism underlying the interaction between Ca and the gut microbiota has not been fully elucidated, a potential pathway has been proposed: *Bifidobacterium* and *Lactobacillus* in the gut produce short-chain fatty acids (SCFAs, primarily butyrate), which lower colonic pH, increase ionic Ca concentration, and promote Ca absorption via passive diffusion through the paracellular pathway (221, 222). This provides a direction for further investigating the Ca absorption-microbiota interaction.

**(2) Mg and the gut microbiota**

Mg is the fourth most abundant cation in the human body (223). As a cofactor for over 300 enzymatic reactions, Mg^2+^ is involved in critical metabolic pathways, including nutrient catabolism, oxidative phosphorylation, DNA and protein synthesis, neuromuscular excitability, and parathyroid hormone secretion (224). The main dietary sources of Mg include nuts, vegetables, and dairy products (225). The intestinal absorption rate of Mg^2+^ ranges from 30 to 50%, with absorption occurring primarily in the small intestine and to a small extent in the colon (226). Understanding of the interaction between Mg and gut microbiota diversity remains limited, with key findings as follows:

- Gommers et al. reported that low gut microbiota diversity was associated with proton pump inhibitor-induced hypomagnesemia and a low-Mg diet, and *Lactobacillus* and *Bifidobacterium* were linked to low-Mg dietary intake (227);
- Mg deficiency in the diet alters the gut microbiota and induces depression-like behavior in mice, though the specific microbiota involved and the relationship between low-Mg diets and depression require further confirmation (228);
- In non-Mg-deficient mice, a low-Mg diet enriched the abundances of *Dorea, Lactobacillus*, and *Turibacter*—microbiota associated with butyrate metabolism (229);

Conversely, a high-Mg diet increased the abundances of *Proteobacteria, Parabacteroides, Butyricimonas*, and *Victivallis* (230). Since Proteobacteria is a key marker of microbiota dysbiosis (231), excessive dietary Mg supplementation may disrupt intestinal microbial balance.

These findings suggest a dose-dependent relationship between dietary Mg intake and the gut microbiota, but additional clinical studies are needed to clarify its physiological regulatory pathways.

**(3) Fe and the gut microbiota**

As a key component of hemoglobin, Fe not only facilitates oxygen transport in the body but also participates in biological pathways such as DNA metabolism and mitochondrial function (232). It also serves as an active-site metal for enzymes like catalase, peroxidase, and cytochrome (233). Dietary Fe exists in heme and non-heme forms, with primary sources including cereals, vegetables, legumes, and fruits. In the small intestine, Fe^2+^ binds to transferrin to form ferritin, which enables Fe absorption (234). Key insights into the Fe-gut microbiota relationship include:

- Ganz and Nemeth noted that Fe intake is associated with immune regulation (235), and Fe deficiency is common in patients with inflammatory bowel disease (IBD) (236, 237). Gut microbiota dysregulation is a well-documented hallmark of IBD (238);
- Das et al. reported that gut microbiota metabolites reduce intestinal Fe absorption by inhibiting hypoxia-inducible factors (239);
- In a colitis mouse model, microbiota-derived valerate restored immune tolerance and alleviated Fe deficiency symptoms by promoting intestinal Fe uptake and regulating regulatory T-cell differentiation (240);
- Probiotic supplementation enhances Fe absorption: *Bifidobacteria* and Lactobacillus lower intestinal pH by producing amino acids or SCFAs, thereby optimizing the bioavailability of dietary Fe (241);

Notably, several studies showed that dietary Fe supplementation reduces the abundances of *Bifidobacteria* and *Lactobacillus* in the infant gut (242–244). Additionally, Fe supplementation was associated with increased calprotectin levels in infants, indicating heightened intestinal inflammation (244). These results suggest that the impact of Fe supplementation on gut microbiota diversity may vary with host age. Currently, studies on host-related factors influencing the Fe-gut microbiota relationship are scarce, and the exact mechanism by which Fe levels alter gut microbiota structure and activity remains unclear, requiring more experimental evidence.

**(4) Zn and the gut microbiota**

Zn is the second most abundant metallic element in the human body (after Fe). It is involved in biological functions such as biomacromolecule synthesis, neurotransmission, hormone release, and regulation of the oxidative cascade and immune system (245, 246). It also acts as a cofactor in enzymatic catalytic processes (247). Dietary Zn is widely available in poultry, seafood, legumes, nuts, whole grains, and small amounts in dairy products (248). Zn absorption occurs throughout the small intestine, primarily in the duodenum and jejunum (249).

Zn exerts its mucosal protective effects by binding to zinc finger transcription factors (e.g., ZNF365) and activating the expression of mucin 2 (MUC2)—the major component of intestinal mucus (250, 251). Additionally, Zn inhibits the TLR4/MyD88/NF-κB pathway in intestinal macrophages: it binds to the TLR4 extracellular domain, preventing LPS binding, and suppresses IκBα phosphorylation, thereby reducing the transcription of pro-inflammatory cytokines (IL-1β, IL-6) (252, 253). In gut microbiota, Zn is a cofactor for bacterial metalloenzymes (e.g., alkaline phosphatases in *Lactobacillus*), which dephosphorylate LPS and reduce its endotoxin activity (254, 255). Zn is essential for the gut microbiota, which absorbs approximately 20% of dietary Zn. However, clinical evidence for the Zn-gut microbiota relationship is limited, with most data from animal studies:

- Massot-Cladera et al. reported that after Wistar rats consumed acacia, the abundances of *Lactobacillus* and *Bifidobacterium* in the gut increased, and this increase was correlated with Zn concentration (254);
- Zn deficiency increased the abundances of *Proteobacteria, Enterobacteriaceae*, and *Enterococcus* in the cecum of red chickens (256);
- Feeding red roosters Zn-biofortified wheat for 6 weeks led to intestinal enrichment of *Lactobacillus* and *Ruminococcus* (255);
- In pregnant mice with acute Zn deficiency, a low-Zn diet increased the abundances of *Actinobacteria, Bacteroidetes*, and *Firmicutes* while decreasing *Proteobacteria*, and induced inflammation and mild behavioral abnormalities in both pregnant mice and their offspring. Supplementation with a Zn-amino acid conjugate partially restored microbiota composition and reduced inflammation (251);
- Zackular and Skaar found that in a *Clostridium difficile* infection mouse model, excessive dietary Zn altered the gut microbiota and reduced resistance to *C. difficile* infection (253).

However, dose-response studies are currently lacking. Further evidence is required to fully elucidate the relationship between zinc intake and the composition of the gut microbiota.

**(5) Se (Selenium) and the Gut Microbiota**

Se is an essential micronutrient with antioxidant, anti-inflammatory, and antiviral properties (257). Se deficiency can cause thyroid, cardiac, and skeletal muscle diseases (258, 259), and low plasma Se levels are associated with impaired cognitive function and neurological disorders (260). Dietary Se is obtained in both organic and inorganic forms, with Brazil nuts, cereals, meat, fish, seafood, and dairy products being the best sources (261). Se absorption primarily occurs in the small intestine (262)—the colon has lower Se absorption due to its low oxygen content (263).

Se exerts its biological effects as a component of selenoproteins, such as glutathione peroxidase (GPx) and thioredoxin reductase (TrxR). In the gut, *Lactobacillus* and *Bifidobacterium* convert inorganic Se to organic Se, which is more bioavailable to the host (264, 265). GPx1, expressed in intestinal epithelial cells, scavenges ROS and prevents lipid peroxidation of the gut barrier. TrxR1 regulates Treg cell differentiation by reducing oxidative stress, promoting IL-10 secretion and suppressing T helper cell 17 (Th17)-mediated inflammation (264, 266). Additionally, Se supplementation increases *Akkermansia muciniphila* abundance, which enhances gut barrier function by upregulating MUC2 and tight junction proteins—this reduces LPS translocation and systemic inflammation linked to depression (264, 267).

Key insights into the Se-gut microbiota relationship include:

- Dietary Se influences host Se status; the gut microbiota regulates selenoprotein expression via dietary Se intake (268), and Se modulates gut microbiota diversity (269);
- Approximately 25% of bacteria possess genes encoding selenoproteins, and some (e.g., *Escherichia coli, Clostridium difficile, Enterobacteriaceae*) can colonize the human gastrointestinal tract (270);
- Trace element comparative genomics has identified Se-rich bacterial groups (*anaerobic Deltaproteobacteria* and *Clostridia*) in ~600 bacterial and archaeal genomes over the past 15 years (271);
- Disease-related Se-microbiota interactions: Zhai et al. (264) reported that dietary Se supplementation reversed DSS-induced colonic inflammation in mice by increasing the abundances of *Turicibacter* and *Akkermansia* (264);
- In a clinical study, Weng et al. found an association between dietary Se and the abundances of *Firmicutes* and *Verrucomicrobia* in IBD patients (267);
- *Lactobacillus* (an important gut bacterial group) can incorporate Se into selenocysteine, increasing Se availability in human cells (265);
- Tamtaji et al. conducted a randomized human trial showing that combining probiotics (containing *Lactobacillus acidophilus, Bifidobacterium bifidum*, and *Bifidobacterium*) with Se supplements improved cognitive function in Alzheimer's patients (266).

These studies confirm a close relationship between dietary Se and the gut microbiota, suggesting Se may have potential for microbiota-mediated disease treatment. Thus, understanding the Se-gut microbiota interaction is of great significance.

### 7.2 Mineral elements - gut microbiota - depression

It is estimated that over 2 billion people worldwide are deficient in key mineral elements (272). Mineral elements have been linked to cognitive function (273), intestinal disorders (274), and mental disorders (275). However, their role in the etiology and progression of depression remains unclear. Based on the aforementioned mineral element-gut microbiota relationships and the role of the gut-brain axis in depression (276, 277), this section analyzes the association between mineral elements and depressive symptoms from the perspective of how dietary mineral intake modulates the gut microbiota.

**(1) Evidence for mineral elements in depression intervention**

Micronutrient supplementation has been investigated as an adjunctive treatment for depression, with Fe, Zn, Mg, and Se being the most studied:

- Postpartum Fe levels are strongly associated with depression and cognitive function, and Fe supplementation may alleviate depression-like symptoms (278);
- Multiple depression treatment trials showed that combining Zn supplements with antidepressants enhanced therapeutic efficacy (279, 280);
- A Mg-rich Mediterranean diet reduced anxiety symptoms and mood disorders in women (281);
- Recent studies demonstrated that Se supplementation significantly alleviated depressive symptoms (282).

**(2) Potential mechanisms: mineral elements** **→ Gut microbiota** **→** **depression**

**Figure 4:**
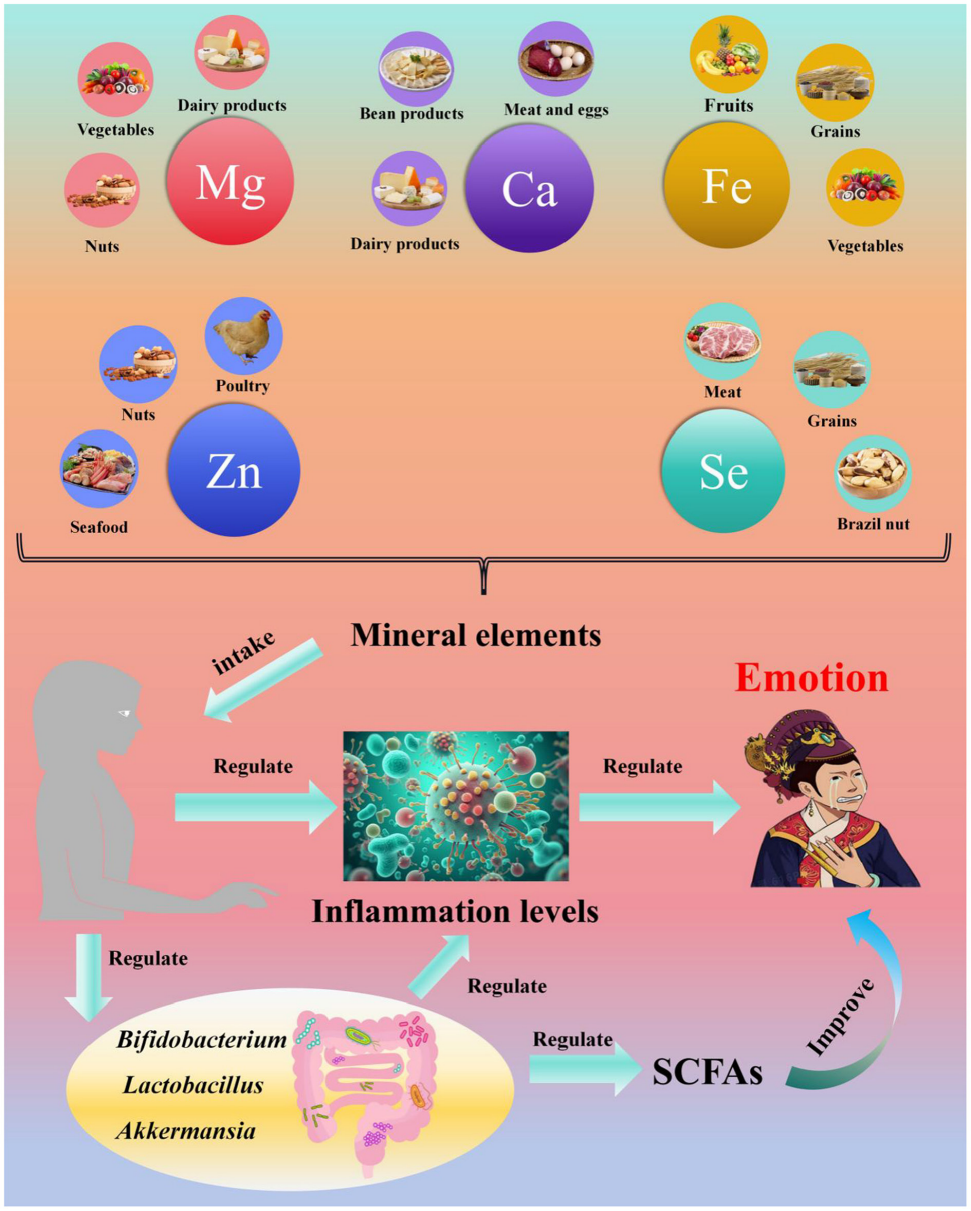
Relationships among minerals, the gut microbiota, and mood. The sources, optimal intake, gut microbiota interactions, and anti-depressant pathways of minerals, along with key notes: it includes specific minerals [zinc (Zn): optimal 15–20 mg/day, from poultry/seafood/legumes; selenium (Se): optimal 50–100 μg/d, from Brazil nuts/fish; iron (Fe): optimal 15–20 mg/day, heme Fe from red meat, non-heme Fe from cereals; calcium (Ca) and magnesium (Mg): from dairy/nuts, with weaker depression evidence], their gut microbiota interactions (Zn/Se promote *Lactobacillus, Akkermansia* and *Faecalibacterium*, Zn upregulating intestinal tight junction proteins, Se enhancing GPx activity; moderate Fe increases Lactobacillus to improve Fe absorption while excess Fe inhibits *Bifidobacterium*; Ca increases *Bifidobacterium* in obese mice, Mg deficiency enriches pro-inflammatory *Proteobacteria*), anti-depressant pathways (Zn/Se reduce LPS-induced inflammation and support SCFA production via beneficial bacteria; Fe maintains tryptophan metabolism to prevent 5-HT deficiency; mineral-regulated microbiota signal to the brain via SCFAs and the vagus nerve to alleviate depressive symptoms), and a note that dotted lines indicate “risk effects” of excessive intake (e.g., >40 mg/day Zn leading to *Enterococcus* overgrowth).

These micronutrients may influence depression through similar biological pathways, with the gut microbiota serving as a key mediator. As highlighted in the preceding sections, mineral elements (Ca, Mg, Fe, Zn, and Se) promote the growth of beneficial gut bacteria, including *Bifidobacterium, Lactobacillus*, and Akkermansia (227, 242, 254, 264); these beneficial microbiota improve depression-like symptoms through two core pathways:

- Producing SCFAs to regulate the gut-brain axis (100–102, 283);
- Inhibiting inflammatory responses (284, 285) (Figure 4).

Further prospective studies are required to:

- Elucidate the precise mechanisms by which mineral elements intervene in depression via the gut microbiota;
- Determine the optimal doses of dietary mineral supplementation for improving depressive symptoms.

### 7.3 Dose-response relationships

Fe: A “U-shaped dose-response relationship” exists between iron and depression:

- When 15–20 mg of elemental iron is supplemented daily, the BDI-II score of patients with postpartum depression decreases by 4.0 points (*P* < 0.01) (278), and the abundance of intestinal Lactobacillus increases by 8% (241);
- When intake is below 10 mg/day, iron deficiency causes tryptophan metabolism disorders [reduced 5-HT synthesis (31)], leading to an increased risk of depression;
- When intake exceeds 30 mg/day, excessive iron inhibits the growth of intestinal Bifidobacterium (242), increases inflammation risk, and instead exacerbates depression.

Zn: the antidepressant effect of zinc depends on a “precise dose”:

- When 15–20 mg of zinc is supplemented daily, the remission rate of depressed patients reaches 65% (when combined with antidepressants) (279, 280), and the expression of intestinal tight junction protein (occludin) increases by 15% (252);
- When intake is below 10 mg/day, zinc deficiency impairs the intestinal barrier;
- When intake exceeds 40 mg/day, excessive zinc increases the abundance of intestinal Enterococcus (256), induces microbiota dysbiosis, and offsets the antidepressant effect.

Se: the antidepressant dose window of selenium is 50–100 μg/day

- When serum selenium levels are ≥120 μg/L, the depression score decreases by 3.2 points (282), and the abundance of intestinal Akkermansia increases by 10% (264);
- When intake is below 50 μg/day, selenium deficiency reduces the activity of selenoproteins (e.g., GPx1) and increases inflammatory levels;
- When intake exceeds 200 μg/day, excessive selenium increases the metabolic burden on the liver and kidneys without providing additional antidepressant benefits.

### 7.4 Association between mineral elements intake and depression, and research limitations

The association between minerals and depression has critical limitations, including weak intervention evidence and unclear mediation by microbiota.

- First, animal studies overshadow human data: the link between Mg deficiency and depression-like behavior (228) comes from mouse models, but human Mg metabolism (e.g., renal excretion) differs, and Mg deficiency in humans is often accompanied by vitamin D deficiency [a confounder not addressed in (228)].
- Second, microbiota mediation is unconfirmed: the original analysis assumes minerals “regulate microbiota to improve depression,” but no RCT has validated that mineral supplementation [e.g., Se (282)] changes microbiota composition (e.g., increases Akkermansia) and subsequently reduces depressive symptoms (264, 282).
- Finally, observational studies cannot rule out reverse causation: low Fe levels (278) may be a result of depression (e.g., depressed patients have poor appetite) rather than a cause, a factor ignored in the original interpretation (278).

## 8 Correlations between dietary patterns and depression

### 8.1 Unhealthy dietary choices, nutritional imbalances, and depression risk

In modern society, individuals face growing levels of stress and often turn to junk foods such as cola, cakes, and potato chips under the perception that these foods can induce temporary feelings of pleasure. While many believe such foods—despite the risk of weight gain—contribute to happiness, this is a misconception. The short-term pleasure derived from an unhealthy diet is quickly followed by a return to low mood or even exacerbated depression over the long term.

### 8.2 Empirical evidence supports the link between unhealthy diets and elevated depression risk

- Kashino et al. conducted a 3-year (2012–2016) study on an occupational cohort of 935 Japanese adults, finding that 16.9% (158 cases) of participants exhibited depressive symptoms. Notably, higher consumption of soft drinks correlated with greater depression risk: compared to individuals who consumed no sweetened beverages per week, those who drank more than four servings of sweetened beverages daily had a 90% increased risk of depressive symptoms (multivariately adjusted OR = 1.91, 95% CI: 1.11–3.29; Ptrend = 0.015) (14).
- Chen et al. followed 126,819 participants from the UK Biobank for an average of 7.6 years. Their findings indicated that two dietary patterns (DPs) were significantly associated with increased risk of depression and anxiety symptoms: DP1 (high in sugar, low in dietary fiber) and DP3 (high in sugar and fat, high in fiber). In contrast, DP2 (high in sugar, low in fat) showed no association with depression or anxiety (15).

### 8.3 The mediterranean diet and depression

Diet plays a critical role in the onset and intervention of psychiatric disorders. Adopting an anti-inflammatory diet—including increased intake of deep-sea fish, adequate consumption of fatty acids (e.g., folic acid) and magnesium, and avoidance of processed foods—has been linked to a reduced risk of psychiatric disorders (286). Thus, selecting an appropriate dietary structure is crucial for the prevention and early intervention of depression. The following sections discuss the relationships between three specific dietary patterns (Mediterranean diet, DASH (Dietary Approaches to Stop Hypertension) diet, and Okinawa diet) and depression.

#### 8.3.1 Definition, characteristics, and nutritional goals of the mediterranean diet

The Mediterranean diet is currently the most extensively studied dietary pattern and is widely recognized as healthy. In 2010, the United Nations Educational, Scientific and Cultural Organization (UNESCO) designated it as an “intangible cultural heritage” of France, Italy, Greece, Spain, and Morocco (287). In recent years, it has been further identified as a dietary pattern rich in protective nutrients, with the potential to prevent a range of diseases (287).

Key characteristics of the Mediterranean diet include (287, 288):

- high intake of vegetables, legumes, fresh fruits, unrefined grains, nuts, and olive oil;
- moderate consumption of fish and dairy products;
- low intake of red meat;
- moderate alcohol consumption.

Its nutritional goals are to increase intake of dietary fiber (from vegetables and fruits), carbohydrates (from whole grains), plant-based protein (from legumes), polyunsaturated fatty acids (from deep-sea fish), and vitamins (e.g., vitamin C from fruits), while reducing intake of fat, alcohol, sodium, and added sugars (from sweets) (289).

#### 8.3.2 Evidence linking the mediterranean diet to reduced depression risk

Most studies have demonstrated a significant negative correlation between adherence to the Mediterranean diet and depression incidence:

- A prospective cohort study of Swedish women investigated the association between Mediterranean diet adherence and risk of clinically diagnosed depression. The results showed that middle-aged women who adhered to the Mediterranean diet had a lower risk of depression in later life (290).
- To evaluate whether Mediterranean diet intervention improves moderate-to-severe depressive symptoms in young men, Bayes et al. conducted a 12-week RCT involving 72 Australian men aged 18–25 years. Participants were randomly assigned to either a Mediterranean diet (MD) intervention group or a usual care group. Assessments—including the Mediterranean Diet Adherence Screener (MEDAS), BDI-II, and quality of life (QoL) measures—were conducted at baseline, week 6, and 12. Compared to the usual care group, the MD intervention group showed significant increases in MEDAS scores (indicating better adherence) and QoL scores, as well as a significant decrease in BDI-II scores (indicating reduced depressive symptoms). These findings highlight the important role of dietary nutrition in depression treatment (291).

#### 8.3.3 Mechanisms underlying the mediterranean diet's antidepressant effects

**Figure 5:**
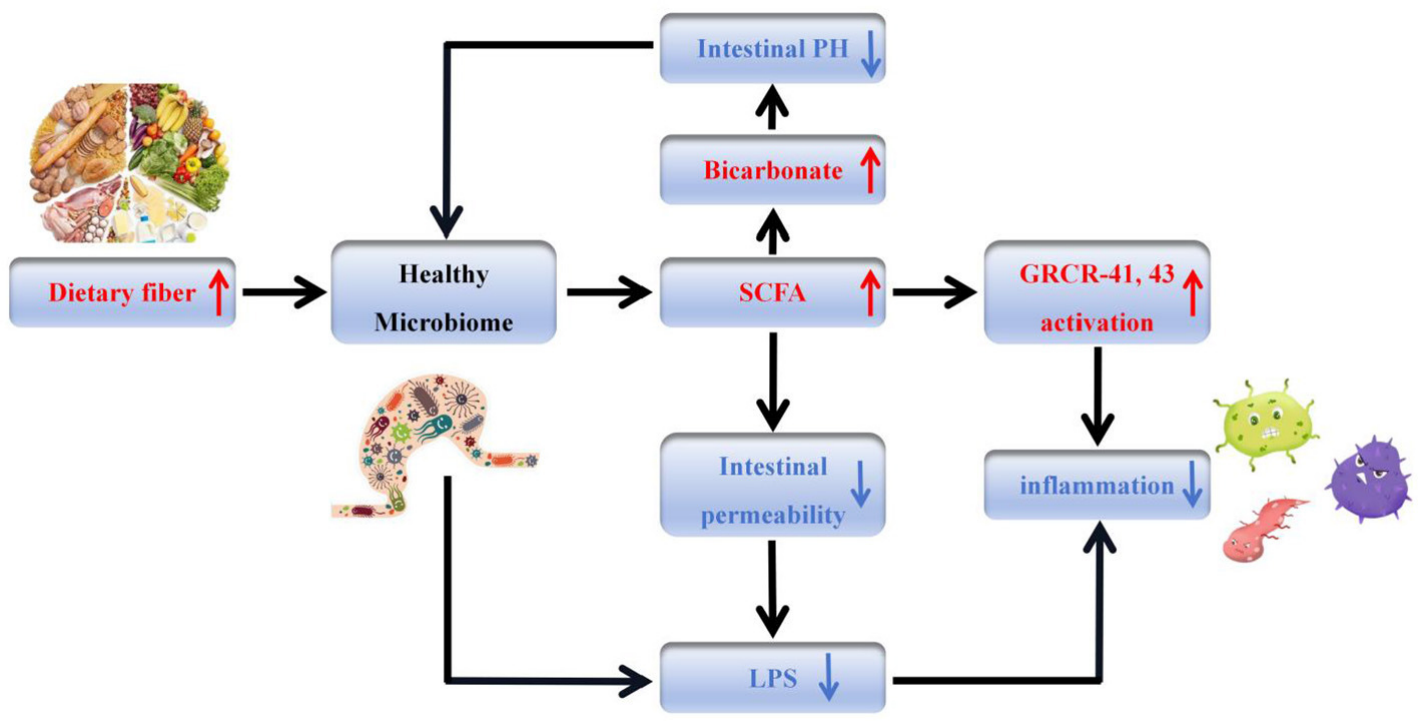
Inflammation is a potential mediator between dietary fiber and depression [modified version of the original picture from the review article by Swann et al. (134)]. The regulatory cascade of dietary fiber on gut microbiota, inflammation, and mood: dietary fiber (e.g., inulin, RS) is fermented by gut microbiota (*Bifidobacterium, Faecalibacterium*) to produce SCFAs (acetate, propionate, butyrate); SCFAs exert anti-inflammatory effects by lowering colonic pH (inhibiting pro-inflammatory bacteria like Enterobacteriaceae and reducing LPS release), binding to GPR41/43 on intestinal epithelial cells (upregulating tight junction proteins to reduce intestinal permeability and suppressing NLRP3 inflammasome activation to decrease IL-1β production), and inhibiting systemic inflammation (weakening brain neuroinflammation by reducing LPS translocation and pro-inflammatory cytokines such as IL-6 and TNF-α); this further inhibits the “fiber deficiency → microbiota dysbiosis → inflammation → depression” cascade, with sufficient fiber intake reducing the risk of depressive symptoms by maintaining gut-brain axis homeostasis (arrows indicate “regulatory direction,” e.g., fiber → SCFA → ↓ inflammation → ↓ depression; “LPS” represents lipopolysaccharide, a key pro-inflammatory signal).

**Figure 6:**
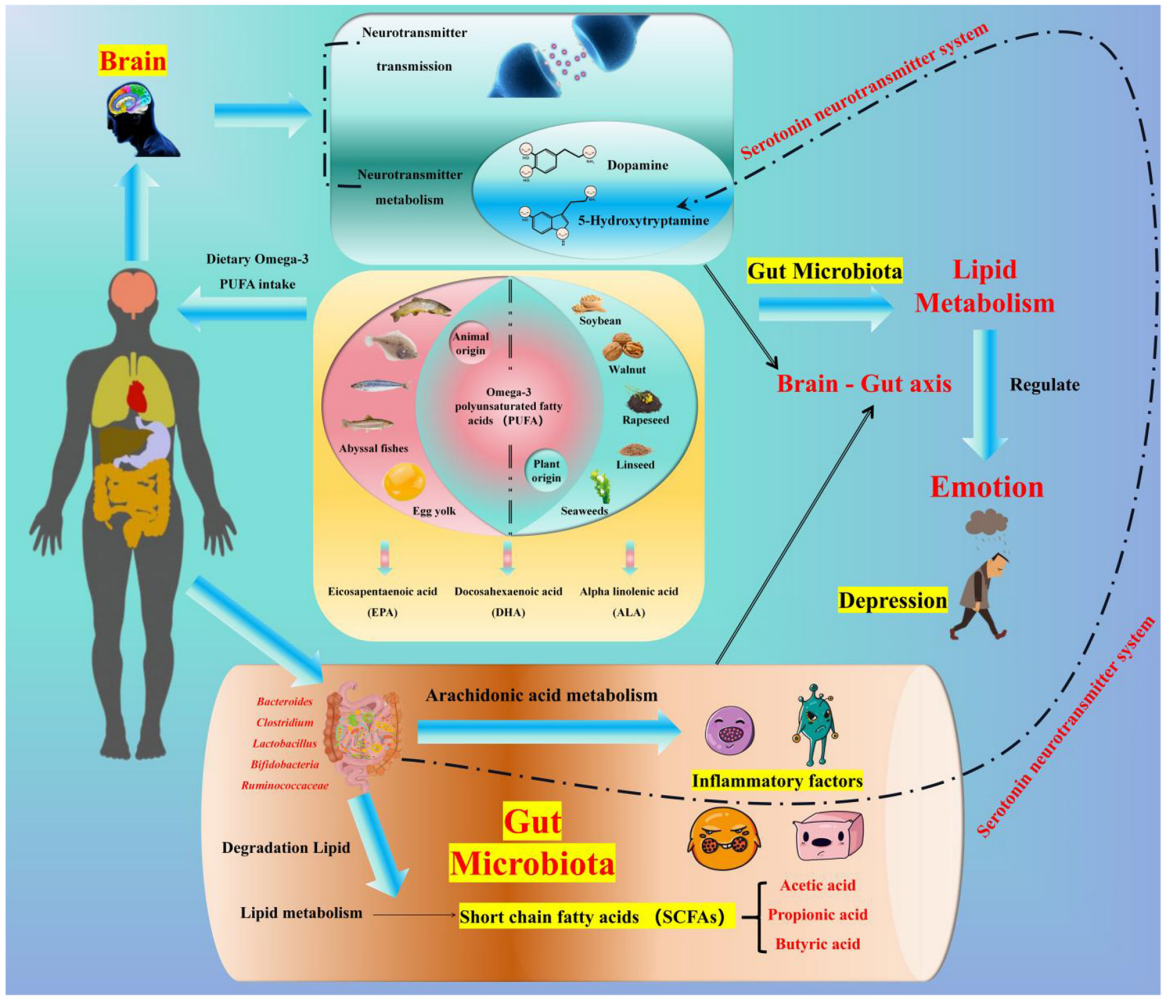
Relationships among omega-3 fatty acids, the gut microbiota, and mood. Omega-3 PUFAs (EPA/DHA from deep-sea fish like salmon, ALA from soybeans and wheat germ—with ALA showing inconsistent effects due to low human conversion efficiency, indicated by a dotted line) interact with gut microbiota (promoting beneficial bacteria such as *Roseburia, Akkermansia*, and *Bifidobacterium*, inhibiting pro-inflammatory bacteria like *Enterobacteriaceae*, which further metabolize omega-3 PUFAs to produce SPMs and SCFAs) to modulate mood and alleviate depression via three anti-depressant pathways: competing with AA for COX/LOX enzymes to reduce pro-inflammatory cytokines (IL-1β, TNF-α) and SPMs inhibiting brain microglial activation (anti-inflammation), DHA enhancing frontal cortex 5-HT2A receptor sensitivity and promoting dopamine transmission (neurotransmitter modulation), and SCFAs restoring HPA axis balance to reduce stress-induced excessive cortisol (gut-brain axis communication).

The antidepressant effects of the Mediterranean diet are primarily mediated by its key components (dietary fiber, polyunsaturated fatty acids, and vitamins) through the following pathways:

Dietary fiber → gut microbiota → SCFAs:

- Dietary fiber acts as a prebiotic to support gut bacterial growth and is fermented by the gut microbiota to produce SCFAs (primarily propionate and butyrate). SCFAs serve as key mediators of the interaction between the Mediterranean diet and gastrointestinal health: they reduce inflammation by enhancing intestinal barrier function (292), alleviate depression-like behaviors, and exert anti-inflammatory and neuroprotective effects (293).
- Inflammation also acts as a potential mediator between dietary fiber and depression, with two proposed regulatory mechanisms: (1) dietary fiber increases SCFA production, which reduces intestinal membrane permeability—thus lowering serum lipopolysaccharide (LPS) levels and suppressing inflammatory responses; (2) dietary fiber modulates inflammation by altering intestinal pH and activating GPRs (Figure 5) (134).
- Omega-3 fatty acids from fish: moderate fish consumption (a hallmark of the Mediterranean diet) provides abundant omega-3 fatty acids, which have demonstrated potential in depression treatment (294). The relationship between omega-3 fatty acids and depression involves three key pathways (Figure 6), as discussed in previous sections.

### 8.4 The DASH diet and depression

#### 8.4.1 Origin, characteristics, and nutritional traits of the DASH diet

The DASH diet was developed in the mid-1990s in response to the rising incidence of hypertension in the United States and is now one of the most widely adopted dietary patterns in the Americas. It emphasizes increased intake of fruits, vegetables, protein, fiber, low-fat dairy products, whole grains, poultry, fish, and nuts, as well as foods rich in blood pressure-lowering minerals (potassium, calcium, and magnesium) (295).

Its core nutritional traits are summarized as follows (296):

- High in potassium, calcium, magnesium, fiber, and protein;
- Low in saturated fat;
- Low in sodium.

#### 8.4.2 Evidence linking the DASH diet to reduced depression risk

Numerous studies have reported a negative association between DASH diet adherence and depression risk:

- A study examining the relationship between DASH diet adherence and mental health in Iranian adults found that moderate adherence to the DASH diet was inversely associated with depression incidence (297).
- A cross-sectional study investigating the associations of the DASH and Mediterranean diets with mental health, sleep quality, and chronotype in overweight and obese women revealed that higher DASH diet adherence was inversely correlated with risk of major depression and extremely high stress scores (298).

#### 8.4.3 Proposed mechanisms and research gaps

Currently, few studies have explored the mechanisms by which the DASH diet reduces depression risk. Proposed hypotheses include:

- Anti-inflammatory effects: the DASH diet is considered an anti-inflammatory dietary pattern. Intake of unprocessed carbohydrates and healthy fats may help reduce systemic inflammation and enhance antioxidant capacity—effects that may contribute to its antidepressant potential (299).
- Mineral-mediated gut microbiota regulation: unlike the Mediterranean diet, the DASH diet prioritizes foods rich in blood pressure-lowering minerals (potassium, calcium, and magnesium). As discussed earlier, calcium and magnesium may improve depressive symptoms by regulating gut microbiota composition and modulating SCFA production and immune function (Figure 4). However, this hypothesis requires further empirical validation.

### 8.5 The Okinawa diet and depression

#### 8.5.1 Definition, core characteristics, and regional context

The Okinawa diet is a traditional Asian dietary pattern centered on root vegetables (primarily sweet potatoes), green and yellow vegetables, soy-based processed foods, and medicinal plants. It also includes moderate intake of marine foods, lean meats, fruits, tea, and wine (300). Its ten defining features are (300):

- Low caloric intake;
- High consumption of vegetables (especially root and green-yellow vegetables);
- High consumption of legumes (mostly soybeans);
- Moderate consumption of fish (higher in coastal areas);
- Low consumption of meat (primarily lean pork);
- Low consumption of dairy products;
- Low total fat intake (with a high ratio of mono- and polyunsaturated fats to saturated fats, and a low omega-6:omega-3 ratio);
- Emphasis on low-glycemic index (GI) carbohydrates;
- High dietary fiber intake;
- Moderate alcohol consumption.

#### 8.5.2 Prevalence of depression in East Asia and the potential of the Okinawa diet

Despite the Okinawa diet's recognition as a healthy pattern, few studies have directly examined its relationship with depression. However, contextual data highlight the need for region-specific dietary interventions for depression:

- In Japan, middle-aged adults are at the highest risk of suicide, and MDD is a key precursor to suicidal behavior. A survey of 362 community residents aged 40–59 years in a rural western Japanese city found an overall depression prevalence of 32.9% (37.1% in men and 29.9% in women) (300).
- In China, mental health risks also persist. A meta-analysis of 40 studies involving 1,024,087 participants reported a current MDD prevalence of 1.1% (95% CI: 0.9%−1.4%), a 12-month prevalence of 1.6% (95% CI: 1.0%−2.5%), and a lifetime prevalence of 1.8% (95% CI: 1.5%−2.2%) (301). While these rates are lower than the global average (12-month MDD prevalence of 5.9%, range: 3.8%−10.4%) reported in the World Mental Health Survey (WMH) (302), China currently lacks a robust community-based psychotherapy system, and patient consultation rates remain low due to stigma (303).

Early dietary intervention may offer unexpected benefits for depression prevention and management. Importantly, the Okinawa diet—like the Mediterranean and DASH diets—adopts an anti-inflammatory framework, emphasizing unprocessed carbohydrates and healthy fats (high in unsaturated fats, low in saturated fats). Thus, it is hypothesized to have antidepressant potential and may be more culturally suitable for Asian populations. However, direct empirical evidence to support this claim is currently lacking.

### 8.6 Key dietary components and depression: dose-effect and modulating factors

#### 8.6.1 Red meat: nutritional contribution, consumption frequency, and depression susceptibility

Red meat plays a dual role in depression, with its effect dependent on nutrient type, processing degree, and weekly consumption frequency—factors that reconcile conflicting findings on its association with mood (30).

**(1) Nutritional contribution to mood regulation**

Unprocessed red meat (e.g., lean beef, lamb) is a critical source of nutrients that support neurotransmitter synthesis and neural function, which are essential for preventing depression-related nutrient deficiencies:

- It provides high-quality protein (20–22 g/100 g) containing all essential amino acids, with a biological availability of >90%—sufficient to meet 25% of daily protein requirements and support tryptophan (serotonin precursor) and tyrosine (dopamine precursor) supply (304).
- It is the primary dietary source of heme iron (1–3.0 mg/100 g), with an absorption rate of 25%−30% (3–5 times higher than plant-derived non-heme iron). Iron deficiency (prevalent in 15% of adults) reduces monoamine oxidase activity, leading to 5-HT degradation and increased depressive symptoms; moderate red meat intake (1–2 servings/week) can reduce iron deficiency-related depression risk by 22% (305).
- It supplies vitamin B12 and Zn: vitamin B12 maintains myelin sheath integrity (preventing prefrontal cortex damage), while zinc modulates TPH1 activity—both deficiencies are associated with higher depression risk (306, 307).

**(2) Dose-effect of weekly consumption frequency**

- High frequency (≥4 servings/week): high meat consumption may be associated with a moderate risk of depression. Even unprocessed red meat at this frequency causes iron overload (inhibiting TPH1 activity) and elevates fecal pro-inflammatory Enterobacteriaceae abundance (308, 309). Processed red meat (e.g., sausages, bacon) exacerbates this effect—each additional serving/week increases serum TNF-α levels and depressive symptom severity (310).
- Moderate frequency (1–2 servings/week): Balances nutritional benefits and inflammation risk, reducing depression risk. This frequency meets iron, B12, and zinc needs without inducing gut dysbiosis, and is associated with higher fecal butyrate levels (vs. high-frequency intake) (309, 311).
- Low frequency (< 1 serving/week): Increases vitamin B12 deficiency risk and neurogenic inflammation, leading to 165% higher depression risk—especially in vegetarians and older adults (312).

#### 8.6.2 Fish and seafood: enhancing depression prevention via multi-nutrient synergy

Fish and seafood are “superfoods” for mood regulation, as they enhance the antidepressant effect of diet through omega-3 PUFAs, vitamin D, and Se synergy—a mechanism more robust than single-nutrient interventions.

**(1) Omega-3 PUFAs (EPA/DHA) as core antidepressant components**

Deep-sea fish (e.g., salmon, mackerel) contain 2–3 g/100 g of EPA and DHA, which target two key pathways of depression:

- **Inhibiting microglial activation:** by inhibiting the NF-κB/MAPK p38 signaling pathway and activating the neuronal BDNF-PI3K/AKT pathway, the process achieves a balance in microglial M1/M2 polarization and provides neuroprotection against neuroinflammation (313).
- **Enhancing neurotransmitter transmission:** DHA accounts for 40% of frontal cortex fatty acids; supplementation increases 5-HT2A receptor sensitivity, improving mood regulation (314).
- **Clinical evidence:** A 12-week RCT of 60 participants showed that a Mediterranean diet rich in fish reduced Depression Anxiety Stress Scales (DASS) stress score by 145%. (315).

**(2) Synergy of vitamin D and Se**

Fish (e.g., sardines) and seafood (e.g., oysters) provide vitamin D (5–10 μg/100 g) and Se (30–50 μg/100 g), which amplify omega-3's effects:

- Vitamin D promotes BDNF expression in the hippocampus, enhancing neuroplasticity and reducing depression recurrence risk (316).
- Se scavenges reactive oxygen species, protecting neurons from oxidative damage induced by nutrient imbalances (e.g., high sugar) (317).

**Combined effect:** At present, there is limited direct research evidence regarding the synergistic effects of Se and vitamin D on depression. However, these two nutrients may exert indirect synergistic influences on depression through shared mechanisms, such as anti-inflammatory and antioxidant activities, as well as the regulation of neuronal function. A study based on data from the 2011–2014 U.S. National Health and Nutrition Examination Survey (NHANES), which included 2,154 adults aged 60 years and older, found that vitamin D, as a mediator related to oxidative stress, significantly mediated the relationship between Se intake and cognitive function, accounting for 8.02% of the association. This suggests a potential synergistic effect of Se and vitamin D in improving cognitive function, whereby Se may positively influence cognition by modulating vitamin D levels (318).

**(3) Gut microbiota modulation**

Phospholipids in fish increase gut *Akkermansia muciniphila* abundance, promoting SCFA production. This synergizes with omega-3 to reduce intestinal permeability (LPS levels down) and strengthen gut-brain axis communication—explaining why fish intake is more effective for depression than omega-3 supplements alone (319).

### 8.7 Individual difference factors modulating diet-depression associations

#### 8.7.1 Age: shaping diet-induced depression progression

Age affects the speed and severity of diet-induced depression by altering gut microbiota diversity, nutrient metabolism capacity, and neuroplasticity—resulting in distinct risk profiles across life stages.

**(1) Adolescence (12–18 years): high sensitivity to high-sugar diets**

Although human studies investigating the gut microbiome in adolescents remain limited, the ongoing development of the gut-brain axis and the adolescent brain suggests that this population may be particularly susceptible to diet-induced depressive symptoms (320),

**The impact of high-sugar diets on metabolic and intestinal health**

- In male C57BL/6J mice, consumption of a high-glucose diet (HGD) over a 12-week period induces hyperglycemia, glucose intolerance, dyslipidemia, and increased adipose deposition. Concurrently, a reduction in the diversity of the intestinal microbiota was observed, characterized by a decrease in the relative abundance of Bacteroidetes and an increase in Proteobacteria. Furthermore, a high-glucose diet enhances intestinal permeability through alterations in tight junction proteins, potentially triggering intestinal inflammation (321).
- Similarly, a high-fructose diet (HFrD) elicits metabolic and microbial changes comparable to those induced by a high-glucose diet. A study (322) demonstrated that male Sprague Dawley rats administered low (2.6 g/kg/day), medium (5.3 g/kg/day), or high (10.5 g/kg/day) doses of fructose over 20 weeks exhibited elevated serum levels of pro-inflammatory cytokines (IL-6 and TNF-α), alongside a reduction in the anti-inflammatory cytokine IL-10. Notably, high fructose intake was also associated with an increased abundance of the genera Parasutterella and Blautia, and a decreased abundance of the genus Intestinimonas.
- In addition, research has shown that feeding male Wistar rats a high-sucrose diet for 4 weeks significantly increases serum triglyceride and cholesterol levels. This metabolic disturbance is accompanied by intestinal microbiota dysbiosis, specifically reflected in the altered ratio of Bacteroidetes to Firmicutes. Specifically, there is an increase in Bacteroidetes and Verrucomicrobiota, along with a decrease in Firmicutes (322).

The daily caloric intake from simple sugar by teenagers is higher than that observed for other age groups [~20% of total (daily caloric intake) (323)], this may increase the risk of depression.

**(2) Adulthood (19–59 years): metabolic resilience and reversibility**

Adults generally maintain a stable dietary pattern, a well-established gut microbiota, and a robust metabolic capacity, which collectively contribute to a greater potential for dietary-related depression to be reversible.

- A cross-sectional study was conducted using the NHANES (2011–2018) database in the United States, which included a total of 18,439 adults aged 20 years and older. Depressive symptoms were assessed using the PHQ-9 questionnaire. Following multivariate logistic regression analysis with adjustment for multiple covariates, the study found that for every 100 g/day increase in dietary sugar intake, the prevalence of depression increased by 28% (OR = 1.28, 95% CI: 1.17–1.40, *P* < 0.001). The findings indicate a positive association between dietary sugar intake and the risk of depression among American adults (324).
- The Epidemiology of Chronic Diseases (EpiDoC) cohort study, which included 10,153 Portuguese adults, employed cluster analysis to identify two predominant dietary patterns: the “meat-based diet pattern” and the “fruit and vegetable-based diet pattern.” After adjusting for multiple confounding variables, the study revealed that men with lower levels of education were more likely to follow a meat-based dietary pattern, which was in turn associated with a higher likelihood of depressive symptoms (325).
- To clarify gut microbiota interventions' efficacy in alleviating depressive symptoms, researchers did systematic reviews and meta-analyses, including RCT on probiotics, prebiotics, synbiotics or fecal microbiota transplantation in adults (≥18 years) that used validated, placebo-controlled measures to assess depressive symptoms. A total of 62 studies formed the final dataset (50 for meta-analysis), and results showed probiotics, prebiotics and synbiotics interventions significantly improved depressive symptoms statistically (326).

**(3) Old Age (≥60 years): reduced adaptability and higher deficiency risk**

Older adults have lower gut microbiota diversity and reduced fiber fermentation efficiency, making even mild nutrient deficiencies impactful:

- A cross-sectional study involving 297 elderly individuals aged 60 years and older (144 males and 153 females) was conducted to assess the relationship between zinc status and the presence of depression or anxiety, using validated scales and diagnostic tools. The results indicated that the overall prevalence of zinc deficiency in this cohort was 23.2%, with 72.4% of participants exhibiting dietary zinc intake levels below the estimated average requirement (EAR). Additionally, the prevalence of depression and anxiety was found to be 42.2 and 52.5%, respectively (327). Concurrently, vitamin B12 deficiency was identified as a significant public health concern, particularly among vulnerable populations such as the elderly and developing embryos, where insufficient B12 levels have been associated with an increased risk of neural tube defects. While strategies such as vitamin supplementation and food fortification are considered promising approaches to mitigate these deficiencies, further research is recommended to develop targeted public health interventions (328).
- Previous studies have demonstrated an association between vitamin B12 deficiency and depression. One clinical trial enrolled 73 eligible participants with normal but low B12 levels and inadequate initial response to selective serotonin reuptake inhibitors (SSRIs), who were randomly assigned to either a treatment group (antidepressants combined with B12 injections, *n* = 34) or a control group (antidepressants alone, *n* = 39). At the 3-month follow-up, 100% of participants in the treatment group exhibited a reduction of at least 20% in their Hamilton Depression Rating Scale (HAM-D) scores, compared to 69% in the control group. These differences remained statistically significant after adjusting for baseline characteristics (all *P* < 0.01). The findings suggest that the combination of B12 supplementation and antidepressants significantly enhances symptom improvement in this patient population (329).
- Another study has emphasized the importance of nutrition for both physical and mental health; however, the causal relationship remains to be fully elucidated. Notably, higher body mass index (BMI) has been linked to a lower risk of depression among older adults, which contrasts with observations in younger populations. A longitudinal cohort study conducted from 2014 to 2017, involving 2,081 individuals aged 65 and above with annual follow-up intervals, revealed a significant reduction in depressive symptoms over time. At baseline, factors such as higher dietary quality, higher BMI, younger age, male gender, and fewer chronic diseases were associated with lower levels of depression. Longitudinally, higher dietary quality, increased BMI, and fewer chronic conditions were also correlated with a decline in depressive symptoms (330).

These results indicate that a high-quality diet may serve as a protective factor against depression in older adults, although further clinical research is warranted to confirm this association.

#### 8.7.2 Ethnicity: diet-microbiota interactions

Ethnicity modulates diet's effect on depression through traditional dietary patterns and gut microbiota divergence—explaining why the same diet has varying efficacy across populations.

**(1) Dietary pattern adaptation**

- Western populations (Europeans, Australians): the Mediterranean diet (rich in omega-3 and olive oil) reduces depression risk, as their gut microbiota (high Faecalibacterium abundance) efficiently metabolizes omega-3 into anti-inflammatory resolvins (86, 87, 331).
- East Asian populations (Chinese, Japanese): the Okinawa diet (high in sweet potatoes, soy protein and Chinese diet (high in soluble fiber) reduce depression risk due to higher Akkermansia abundance and stronger fiber fermentation capacity (SCFA production higher) (16).

**(2) Gut microbiota divergence**

The place of birth significantly influences an individual's gut microbiome, rather than the human species itself. This suggests that dietary factors play a crucial role in shaping the composition of the gut microbiota.

- **Differences in gut microbiota between foreign-born populations and American-born whites:** the abundance ratio of *Bacteroides* to *Prevotella* in foreign-born Korean and Hispanic individuals is lower than that observed in American-born whites. This is primarily due to a higher abundance of *Prevotella copri* within the *Prevotella* genus. Additionally, these foreign-born groups exhibit enrichment of *Bifidobacterium, Paraprevotella*, and *Prevotella*. Specifically, foreign-born Koreans show enrichment of the algal-degrading bacterium *Bacteroides plebeius* and a depletion of the *Rikenellaceae* family. Foreign-born Spaniards demonstrate enrichment of lactic acid bacteria and a depletion of Bacillus catarrhalis. These characteristics are not present in American-born whites (17).
- **Differences in gut microbiota among U.S.-born ethnic groups:** compared with U.S.-born whites, U.S.-born Black individuals show enrichment of *Bifidobacterium* and *Prevotella*, a pattern partially consistent with the differences observed between foreign-born populations and U.S.-born whites. U.S.-born Black and Hispanic individuals exhibit similar microbial patterns at the s-OTU level; however, these patterns differ from those observed between foreign-born groups and U.S.-born whites (17).
- **Microbial changes associated with dietary cultural adaptation:** among foreign-born Koreans and Hispanics, individuals with high levels of dietary adaptation exhibit intestinal abundances that increasingly resemble those of U.S.-born whites. For example, in foreign-born Koreans with high dietary adaptation, the algal-digesting bacterium *Bacteroides plebeius* is depleted. Additionally, among foreign-born Koreans, the *Bilophilas* which is associated with the consumption of high-fat seasonings, is more abundant in those with greater dietary adaptation (17).

**(3) Nutritional intake differences**

Based on the analysis of data from 4,747 adult respondents in the NHANES dataset, significant differences were observed in nutritional intake patterns across different ethnic groups. For instance, Asians exhibited higher intakes of dietary fiber and protein, along with lower intakes of total sugar and fat, whereas whites and blacks demonstrated higher total fat intake and lower protein consumption. Following adjustment for demographic variables, the association between nutrient intake and depressive symptoms was found to vary according to cultural background. Specifically, the ratio of total fat and protein to energy intake was significantly linked to depressive symptoms in Hispanics, dietary fiber to sugar ratio in whites, total energy intake in blacks, and total sugar to dietary fiber ratio in Asians. These findings support and extend existing research on the relationship between dietary patterns and the risk of depression. Furthermore, they suggest that demographic characteristics and immigration-related factors may also influence this association (332).

### 8.8 Non-pharmacological synergies: mind-body medicine

Mind-body medicine (MBM, e.g., mindfulness, yoga, Cognitive Behavioral Therapy (CBT) enhances dietary interventions by reducing stress, improving nutrient absorption, and modulating gut microbiota—offsetting the depressive effects of poor diet (e.g., high sugar, low fiber).

**(1) Stress reduction and gut barrier protection**

- A study compared 12 healthy vegan subjects with long-term meditation practice to 12 healthy omnivorous subjects without meditation experience. The results indicated a significant alteration in the gut microbiota structure of the meditation group, with 14 dominant bacterial genera identified as potential biomarkers for distinguishing the two groups (AUC = 0.92). Notably, three beneficial genera—Bifidobacterium, Roseburia, and Lactobacillus—were significantly enriched in this group. Furthermore, functional pathways associated with flavonoid biosynthesis and antigen processing and presentation were enhanced, while metabolic pathways related to tyrosine and other compounds were reduced. These findings suggest that long-term meditation combined with a vegan diet may positively influence human immunity, regulate endocrine and metabolic functions, and contribute to the maintenance of overall health (333).
- A 6-week randomized, double-blind, placebo-controlled trial involving 31 IBS patients, who were divided into three groups—yoga plus probiotics (YPP), yoga plus placebo (YPPB), and probiotics only (P) —revealed that cardiovascular endurance improved and levels of Klebsiella decreased in the YPP group. Both the YPP and YPPB groups exhibited significant improvements in IBS-specific quality of life scores. The study concludes that the integration of yoga, mindfulness, and probiotics offers comprehensive benefits for individuals with IBS, positively affecting physical fitness, psychological wellbeing, and gut microbiota (334).
- Based on evidence that circular meditation (CM) can enhance vagal tone and improve cognitive function, it is hypothesized that CM may modulate the gut microbiota via the CNS and autonomic nervous system (ANS) pathways, thereby enhancing the communication within the gut-brain axis. Although direct evidence regarding the specific impact of CM on the gut microbiota remains limited, existing studies indicate a positive correlation between meditation practices and the abundance of beneficial bacterial genera such as ^*^Rosiella^*^. Consequently, long-term CM may contribute to a healthier intestinal microbiota, potentially improving gut-brain communication through multiple mechanisms, including microbiota regulation (335). Nevertheless, the current understanding of the potential association between CM and the gut-brain axis (GBA) is based solely on indirect inferences drawn from existing literature. Further systematic research is required to establish a definitive relationship among these three components.

**(2) Improving nutrient absorption**

Research articles examining the impact of psychosomatic medicine on nutrient absorption have not yet been compiled. However, a related concept—mindful eating—has been identified, which integrates mental and physical health through dietary practices. Mindful eating is grounded in the interplay between neurogastrointestinal physiology and stress regulation. By activating the parasympathetic nervous system and assisting patients in recognizing the relationship between stress and dietary habits—such as through the Mindful Eating Questionnaire (MEQ) and handwritten dietary journals—it helps reduce stress responses and enhance digestive function, thereby promoting more favorable conditions for nutrient absorption. Furthermore, as a non-standardized intervention, it allows for personalized implementation tailored to individual patient needs. Mindful eating thus represents a scientifically grounded and effective approach to optimizing digestion and improving overall health (336).

### 8.9 Evidence strength summary of nutrients

The “Nutrient-Gut Microbiota Inhibition” comprehensive evidence table systematically summarizes the evidence regarding the associations between five core nutrient categories—protein, Omega-3 polyunsaturated fatty acids, dietary fiber, vitamins, and minerals—and the “Gut Microbiota Inhibition” axis, as detailed in Sections 3–7 of the manuscript (Table 2). The primary objective is to enable horizontal comparisons of the strength of evidence, key mechanistic findings, and inherent limitations across different nutrients. This synthesis addresses the need for a concise summary of dispersed data and clarifies which nutrients demonstrate robust clinical and mechanistic support for their role in depression intervention via gut microbiota modulation, and which require further investigation. It also lays the groundwork for the subsequent discussion on clinical implications and research gaps presented in Section 9. Key findings from Table 2 include the following: dietary fiber (NSPs/RS) and Omega-3 (EPA/DHA) exhibit strong or high evidence levels, supported by consistent, low-bias human RCT. Both nutrients exert antidepressant effects through gut microbiota regulation, either by enhancing SCFA production or reducing inflammation, making them the most promising candidates for clinical translation (consistent with Sections 4.3, 5.3). Protein (milk- or plant-based) and minerals (Zn/Se) are categorized as having moderate evidence, indicating potential but necessitating further targeted RCT—for instance, the efficacy of protein depends on bioavailability and an intake threshold of 1.2 g/kg body weight/day (27, 28), while zinc dosage should be maintained at 15–20 mg/day (252, 279) to avoid microbiota disruption. Evidence for vitamin E/K and minerals such as iron, calcium, and magnesium remains weak or very low, with insufficient human data. For example, vitamin K research has been largely limited to patients with Crohn's disease (185), and iron's effects vary by age group [infants vs. adults (242)], underscoring the importance of population-specific investigations. The findings compiled in this table directly inform Section 9 (“Discussion”) in three key areas: regarding clinical significance, it supports the prioritization of stratified interventions based on nutrients with strong evidence (e.g., dietary fiber and EPA/DHA), particularly for inflammatory depression subtypes (65, 124); concerning research gaps, it identifies critical areas requiring further exploration, such as developing dose-response models for the “nutrient-microbiota inhibition” axis and investigating potential synergistic effects among nutrients; finally, in terms of limitations, it highlights the heterogeneity in evidence [e.g., variations in Omega-3 dosing across RCTs (65)] and population-specific biases [e.g., vitamin D studies focusing on IBD patients (169)].

**Table 2:** Evidence strength of nutrients.

**Nutrient category**	**Key subtypes/ components**	**Evidence strength**	**Core findings linked to gut microbiota and depression**	**Limitations/confounding factors**	**Citation support**
Proteins	Milk/plant-derived protein; Red/processed meat	Moderate	Milk/plant protein intake reduces depression risk via gut microbiota-regulating tryptophan → 5-HT synthesis. Red/processed meat may increase depression via elevated *Bacteroides*	Plant protein effects vary by region [grain vs. bean subtypes (29)]. Animal studies lack human psychosocial factors (37)	(23, 27–30, 37)
Omega-3 polyunsaturated fatty acids (PUFAs)	EPA; DHA; ALA	Strong (EPA/DHA); Weak (ALA)	EPA/DHA supplementation improves depression via gut microbiota (e.g., Roseburia) reducing pro-inflammatory cytokines (65, 66, 103). - ALA shows no consistent effect [low human conversion efficiency (52)]	RCT heterogeneity [dose: 0.2–3 g/d (65)]. Publication bias in positive results (66)	(52, 65, 66, 103)
Vitamins	Vitamin D; B-group (B6/B12); Vitamin A/C; Vitamin E/K	High (D/B-group); Moderate (A/C); Very Low (E/K)	Vitamin D increases *Akkermansia*/*Roseburia* to alleviate depression. B6/B12 promote gut SCFA production via *Faecalibacterium*. Vitamin E/K lack direct depression-microbiota evidence	Vitamin D studies focus on IBD patients (limited generalizability). Vitamin K data from small Crohn's cohorts	(103, 144, 148, 169, 170, 178, 185)
Minerals	Zinc (Zn); Selenium (Se); Iron (Fe); Calcium (Ca)/Magnesium (Mg)	Moderate (Zn/Se); Weak (Fe/Ca/Mg)	Zn (15–20 mg/day) upregulates gut tight junctions and *Lactobacillus*. Se increases Akkermansia to reduce inflammation. Fe/Ca/Mg lack human microbiota-depression RCTs	Zn excess (≥40 mg/d) disrupts gut microbiota. - Fe effects vary by age (infant vs. adult)	(218, 227, 242, 252, 256, 264, 279, 282)
Dietary fiber (sugars subcategory)	Non-starch polysaccharides (NSPs); Resistant starch (RS)	Strong	NSPs/RS increase *Bifidobacterium*/*Faecalibacterium* to produce SCFAs (butyrate) and enhance gut barrier. Low fiber intake correlates with depressed patients' reduced acetate/propionate	Fiber effects depend on gut microbiota composition (low *Faecalibacterium* = non-response). Confounded by low added sugar intake	(20, 24, 102, 111, 124)

Evidence Strength Definition: Strong: Consistent human RCTs with low bias + clear microbiota-depression mediation; Moderate: Mixed human observational/RCTs + plausible animal mechanistic support; Weak: Limited human data + reliance on animal studies; Very Low: Isolated small studies + no direct depression-microbiota links. All findings and limitations are derived exclusively from revise-manuscript.docx and its cited references.

## 9 Discussion

Insufficient nutrition, a single dietary structure, and long-term excessive consumption of junk food have gradually become key factors inducing the occurrence and progression of depression (14, 15, 286). Metabolites produced by the gut microbiota during growth play crucial roles in maintaining intestinal barrier integrity, facilitating host nutrient absorption, balancing the intestinal microenvironment, and regulating metabolism and immunity (337). Currently, the number of patients with depression is increasing rapidly; however, challenges such as delayed early diagnosis and intervention, poor efficacy of antidepressants, and numerous side effects make depression difficult to detect and treat (4, 6, 338). Thus, there is an urgent need for more non-pharmacological approaches for the early management of depression. Exploring the relationships among nutrients, the gut microbiota, and depression, and promoting optimized dietary patterns, has therefore become a primary strategy for the early intervention of depression in at-risk individuals.

This review summarizes current evidence regarding the potential role of nutrients in modulating the gut microbiota to intervene in depression. Our core objective is to highlight how dietary nutrient intake can improve the structure and diversity of the gut microbiota, thereby enabling early prevention and intervention of depression. Dietary nutrient intake is a critical aspect of human health maintenance and disease intervention. Dietary therapy has garnered increasing attention from researchers due to its multiple health benefits and fewer side effects compared to pharmaceutical treatments (339), particularly in psychiatric disorders—especially within the field of neurogastroenterology (7). The gut microbiota thus serves as a key link between dietary nutrient intake and mood regulation. Current research has focused on dietary supplementation, modulation of the microbiota-gut-brain axis, and combination therapies with probiotics to treat psychiatric disorders; however, most studies in this area remain in the preclinical stage. Most patients with depression exhibit significant nutrient deficiencies. For instance, preclinical and clinical studies have demonstrated that patients with depression lack vitamins and micronutrients, and oral supplementation with multivitamins containing calcium, magnesium, and zinc can alleviate depression-like symptoms (340–342). Furthermore, nutrients are involved in multiple physiological pathways underlying depression (7, 343). From the perspective of the gut microbiota, this review explains that nutrients can enhance the abundance and diversity of the gut microbiota, reduce inflammation, and ultimately improve depression by regulating the gut-brain axis, promoting the synthesis of SCFAs, and modulating neurotransmitter production. Notably, *Bifidobacterium, Lactobacillus, Akkermansia*, and certain butyrate-producing bacteria play pivotal roles in the gut-brain axis.

### 9.1 Integrated discussion on mechanistic evidence strength, gaps, and confounding factors

**(1) Strength ranking of core mechanistic evidence and prioritization for clinical translation**

Based on a systematic analysis of each nutrient's regulatory pathway, the mechanisms with the strongest evidence for clinical translation are prioritized as follows:

- Omega-3 polyunsaturated fatty acids (EPA/DHA) → anti-inflammatory pathway: Supported by human RCTs (65, 69), which show that EPA/DHA supplementation reduces the levels of pro-inflammatory cytokines (IL-1β, IL-6) and improves depressive symptoms. A clear dose-response relationship has been established: ≥1 g/day of EPA is required to exert effects on moderate depression (66). This mechanism holds the highest translational value due to consistent human data and well-defined molecular targets [e.g., competition with arachidonic acid for COX/LOX enzymes (84)].
- Dietary fiber → gut microbiota → SCFA pathway: Validated by human intervention studies (20, 124), such as trials showing THAT inulin supplementation increases fecal butyrate levels and reduces scores on the BDI-II. Mechanistic studies further confirm that SCFAs enhance intestinal barrier function (44) and hippocampal neurogenesis (23). Given the low cost and high accessibility of fiber-rich foods, this mechanism is well-suited for population-wide depression prevention.
- Zinc → intestinal barrier → anti-inflammatory pathway: human RCTs (252) demonstrate that zinc supplementation (20 mg/day) upregulates the expression of intestinal tight junction proteins (e.g., occludin) and reduces systemic inflammation. Consistent results have been observed in both patients with depression and those with IBD (344–346).

In contrast, mechanisms supported by weak or preliminary evidence [e.g., the vitamin C/gut microbiota/depression axis (152, 153), and the calcium/gut microbiota/depression axis (218–220)] require further validation. These mechanisms rely primarily on small-sample studies or animal models and lack *in vivo* mechanistic data from human subjects.

**(2) Cross-nutrient common mechanistic gaps and future research directions**

Across all nutrient categories, three critical gaps in mechanistic understanding persist and must be addressed in future research:

- **Quantification of “microbiota-metabolite-depression” thresholds:** for example, the minimum amount of dietary fiber required to elevate fecal butyrate concentrations to levels that alleviate depressive symptoms remains unclear. Current estimates range from 25 to 30 g/day (124), but this has not been validated in populations with depression. Similarly, the minimum abundance of *Akkermansia* or *Bifidobacterium* needed to exert antidepressant effects has not been defined, which limits the development of targeted microbiota interventions.
- **Bidirectional interactions between nutrients and the gut microbiota:** most studies focus on the unidirectional effect of “nutrients regulating the microbiota” [e.g., vitamin D increasing the abundance of Roseburia (103)]. However, the reverse interaction—“the microbiota modulating nutrient bioavailability” [e.g., gut bacteria synthesizing B-group vitamins (144) or converting inorganic selenium to organic selenocysteine (265)]—has not been linked to depression. This bidirectionality may explain why some nutrient interventions fail [e.g., vitamin B12 supplementation is ineffective in patients with gut microbiota dysbiosis (149)].
- **Synergistic effects of multiple nutrients:** this review addresses nutrients in isolation; however, real-world diets involve nutrient combinations [e.g., the Mediterranean diet, which combines fiber, Omega-3 fatty acids, and B-group vitamins (287–289)]. It remains unknown whether these nutrients act synergistically [e.g., fiber enhancing Omega-3 absorption (294)] or antagonistically [e.g., high calcium intake potentially reducing iron absorption (221)]. This gap limits the design of effective dietary patterns for depression prevention and intervention.

**(3) Prevalent potential confounding factors and recommendations for study design optimization**

Several confounding factors consistently obscure the causal links between nutrients, the gut microbiota, and depression. Future studies should adopt targeted designs to mitigate these issues:

- **Confounding by coexisting nutrient deficiencies:** mineral deficiencies [e.g., zinc (344)] or vitamin deficiencies [e.g., vitamin D (169, 347–349)] often co-occur with protein or fiber deficiencies. For example, individuals with iron deficiency are more likely to have low vitamin C intake (241), which impairs iron absorption. This means that observations such as “iron supplementation improves depression (278)” may actually reflect the effect of vitamin C. Future studies should measure multiple nutrients simultaneously and use multivariate models to isolate the independent effects of individual nutrients.
- **Confounding by lifestyle factors:** high intake of beneficial nutrients [e.g., Omega-3 fatty acids from fish (5, 350, 351)] is often associated with other healthy behaviors [e.g., regular physical activity, low alcohol consumption (286)]. Observational studies (27, 290) fail to fully adjust for these factors, leading to overestimation of nutrient effects. RCTs with rigorous lifestyle control [e.g., standardized physical activity protocols] are needed to confirm causal relationships.
- **Confounding by supplement excipients:** many nutrient supplements [e.g., calcium carbonate (221), zinc gluconate (254)] contain excipients [e.g., lactose, maltodextrin] that can independently alter gut microbiota composition. For example, lactose may increase *Bifidobacterium* abundance (221), which could be misattributed to the effect of calcium. Future intervention studies should use placebo-controlled designs with identical excipients to eliminate this bias.

However, this review has several limitations. First, it does not clearly describe the relationships among dietary nutrient intake, bacterial availability, and depressive symptoms. Second, excessive mineral intake can also reduce gut microbiota abundance; yet, information on the relationship between mineral intake levels and the gut microbiota remains scarce, and insufficient research currently limits our ability to clarify this relationship. Additionally, research on the interactions between the gut microbiota and nutrients is still inadequate, and the specific mechanisms underlying these interactions remain unclear. Despite these limitations, this review highlights that dietary nutrients can modulate gut microbiota composition and alleviate depression-like symptoms. A growing body of evidence supports the need to explore links between dietary factors and mental disorders. Future research should bridge the gap between nutritional neuroscience and clinical evidence, optimize overall dietary patterns, and investigate the mechanisms by which nutrients interact with the gut microbiota and central nervous system—ultimately improving and preventing mental health through dietary interventions.

## 10 Conclusion

This review systematically analyzes the mechanisms, clinical evidence, and research gaps underlying how nutrients improve depression by regulating gut microbiota. Gut microbiota acts as a common mediator for key nutrients [e.g., fiber, Omega-3, zinc], which exert antidepressant effects via shared pathways (enhancing beneficial bacteria, promoting SCFAs, reducing inflammation, regulating the gut-brain axis) and nutrient-specific regulation [e.g., Omega-3 enriching Roseburia, fiber boosting SCFA-producing bacteria]. Healthy dietary patterns (Mediterranean, DASH diets) outperform single-nutrient supplementation due to synergistic nutrient effects. Age and ethnicity influence intervention responses: adolescents are sensitive to diet-induced dysbiosis, the elderly need higher nutrient doses, Western populations benefit more from Omega-3, and East Asians from fiber. Key gaps include limited human RCTs for mechanism validation, overlooked microbiota-nutrient bidirectional interactions, and lack of personalized strategies. In conclusion, nutrients provide safe non-pharmaceutical depression interventions [e.g., 2 weekly oily fish, 25–30 g/day fiber]. Future research should focus on mechanism quantification and precision to translate findings into practice and reduce global depression burden.

## GLOSSARY

SCFA, short-chain fatty acid; ALA, alpha-linolenic acid; MDD, major depressive disorder; CNS, central nervous system; ENS, enteric nervous system; PRISMA, Preferred Reporting Items for Systematic Reviews and Meta-Analyses; CNKI, China National Knowledge Infrastructure; RCTs, Randomized controlled trials; LAT1, L-amino acid transporter 1; ECs, Enterochromaffin cells; GCPRs, G protein-coupled receptors; HDACs, histone deacetylases; EPA, eicosapentaenoic acid; DHA, docosahexaenoic acid; LC-PUFAs, long-chain polyunsaturated fatty acids; AA, arachidonic acid; PLA2, phospholipase A2; PGE2, Prostaglandin E2; COX, cyclooxygenase; LOX, lipoxygenase; SPMs, specialized pro-resolving mediators; RvE1, resolvin E1; TLR4, toll-like receptor 4; IL-1β, Interleukin-1β; NSPs, Non-starch polysaccharides; RS, Resistant starch; RIOS, Resistant/indigestible oligosaccharides; MU, monomer unit; HMW, high-molecular-weight; MCT1, monocarboxylate transporter 1; HPA, hypothalamic-pituitary-adrenal; BL, Bifidobacterium longum NCC3001; IBS, irritable bowel syndrome ROS, reactive oxygen species; DSS, dextran sulfate sodium; RDA, Recommended Daily Allowance; PLP, pyridoxal 5'-phosphate; KAT, kynurenine transaminase; KP, kynurenine pathway; NAD+, nicotinamide adenine dinucleotide; NMDA, N-methyl-D-aspartate; CD, Crohn's disease; RXR, retinoic acid X receptor; VDREs, vitamin D response elements; AMPs, antimicrobial peptides; Zn, zinc; Ca, calcium; Fe, iron; Mg, Magnesium; IBD, inflammatory bowel disease; ZNF, zinc finger transcription factors; MUC2, mucin 2; LPS, Lipopolysaccharides; GPx, glutathione peroxidase; BDI-II, Beck Depression Inventory-II; DPs, dietary patterns; UNESCO, United Nations Educational, Scientific and Cultural Organization; MD, Mediterranean diet; MEDAS, Mediterranean Diet Adherence Screener; QoL, quality of life; GI, glycemic index; DASS, Depression Anxiety Stress Scales; NHANES, National Health and Nutrition Examination Survey; HGD, high-glucose diet; HFrD, high-fructose diet; EAR, estimated average requirement; SSRIs, selective serotonin reuptake inhibitors; HAM-D, Hamilton Depression Rating Scale; BMI, body mass index; MBM, Mind-body medicine; CM, circular meditation; ANS, autonomic nervous system; GBA, gut-brain axis; MEQ, Mindful Eating Questionnaire; TPH1, Tryptophan hydroxylase 1; TPH2, tryptophan hydroxylase 2; PHQ-9, Patient Health Questionnaire-9; PUFAs, Polyunsaturated fatty acids; FOS, Fructo - OligoSaccharide; NLRP3, NLR family pyrin domain containing 3; CD14, Cluster of Differentiation 14; GPR43, G protein-coupled receptor 43; Th17, T helper cell 17; YPP, yoga plus probiotics; YPPB, yoga plus placebo; P, Probiotics.
